# Increased hepatic glucose production with lower oxidative metabolism in the growth-restricted fetus

**DOI:** 10.1172/jci.insight.176497

**Published:** 2024-04-30

**Authors:** Laura D. Brown, Paul J. Rozance, Dong Wang, Evren C. Eroglu, Randall B. Wilkening, Ashley Solmonson, Stephanie R. Wesolowski

**Affiliations:** 1 Department of Pediatrics, School of Medicine, University of Colorado Anschutz Medical Campus, Aurora, Colorado, USA.; 2 University of Texas Southwestern Medical Center, Dallas, Texas, USA.

**Keywords:** Reproductive biology, Gluconeogenesis

## Abstract

Fetal growth restriction (FGR) is accompanied by early activation of hepatic glucose production (HGP), a hallmark of type 2 diabetes (T2D). Here, we used fetal hepatic catheterization to directly measure HGP and substrate flux in a sheep FGR model. We hypothesized that FGR fetuses would have increased hepatic lactate and amino acid uptake to support increased HGP. Indeed, FGR fetuses compared with normal (CON) fetuses had increased HGP and activation of gluconeogenic genes. Unexpectedly, hepatic pyruvate output was increased, while hepatic lactate and gluconeogenic amino acid uptake rates were decreased in FGR liver. Hepatic oxygen consumption and total substrate uptake rates were lower. In FGR liver tissue, metabolite abundance, ^13^C-metabolite labeling, enzymatic activity, and gene expression supported decreased pyruvate oxidation and increased lactate production. Isolated hepatocytes from FGR fetuses had greater intrinsic capacity for lactate-fueled glucose production. FGR livers also had lower energy (ATP) and redox state (NADH/NAD+ ratio). Thus, reduced hepatic oxidative metabolism may make carbons available for increased HGP, but also produces nutrient and energetic stress in FGR liver. Intrinsic programming of these pathways regulating HGP in the FGR fetus may underlie increased HGP and T2D risk postnatally.

## Abstract 2

A sheep model of fetal growth restriction is characterized by reduced hepatic oxidative metabolism, which may make carbons available for increased hepatic glucose production but also produces nutrient and energetic stress.

## Graphical abstract

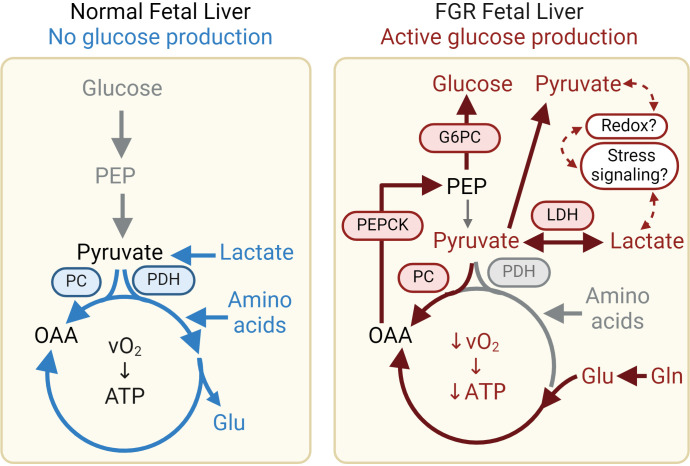

## Introduction

During fetal growth restriction (FGR), the liver develops metabolic adaptations that are beneficial for the fetus, yet maladaptive postnatally. For example, hepatic glucose production (HGP) is not active in the normal fetus because the glucose is supplied in sufficient quantities from the mother across the placenta (1–3). However, the FGR fetus has a decreased glucose supply and an early activation of endogenous glucose production that is resistant to suppression with insulin (4, 5). In addition, human newborns born preterm or with FGR (6–9) and postnatal offspring from rodent models with FGR (10, 11) demonstrate persistently increased and dysregulated glucose production. Importantly, dysregulated HGP is a hallmark of type 2 diabetes (T2D) and FGR offspring have an increased risk of developing T2D (12–14). Thus, early events initiating dysregulated HGP may underlie T2D risk postnatally. However, the mechanisms regulating increased HGP in the FGR fetus are poorly understood.

Gluconeogenic flux is regulated by the activity of cytosolic phosphoenolpyruvate carboxykinase (PEPCK-C, *PCK1* gene), mitochondrial PEPCK (PEPCK-M, *PCK2*), glucose-6-phosphatase (G6Pase, *G6PC*), and pyruvate carboxylase (*PC*). Our prior data demonstrate increased hepatic expression of these genes and protein abundance of PEPCK-C and G6Pase in FGR compared with normally growing control fetal sheep (4). This supports the idea that the liver is likely the major organ contributing to the increased endogenous glucose production; however, this has previously only been measured indirectly using whole-body-based tracer methods (4). Gluconeogenesis requires two 3-carbon precursors (lactate, pyruvate, or alanine) and energy substrates (4 ATP, 2 GTP, and 2 NADH). Indeed, lactate concentrations are increased in FGR human and sheep fetuses (4, 15–17), and FGR fetal sheep and postnatal rats have evidence of limited hepatic glucose (pyruvate) oxidation and increased lactate production (10, 11, 18–20). However, umbilical nutrient supply, whole-body oxidative metabolism, and skeletal muscle ATP content are decreased, suggesting a lower availability of energy substrates in the FGR fetus (4, 15, 18, 19, 21–23). Thus, while prior studies support increased HGP, direct measurements of hepatic glucose output and carbon and energy substrate availability have not been performed in the FGR fetus.

Direct catheterization of the hepatic circulation can be performed in fetal sheep and has demonstrated that the fetal liver, under normal conditions, utilizes amino acids and lactate to fuel oxidative metabolism, with only a small net uptake of glucose (3, 24–26). Moreover, there are 3 reciprocal shuttles between the fetal liver and placenta whereby the liver takes up lactate, glutamine, and glycine from the placenta, and releases pyruvate, glutamate, and serine back to the placenta. In the fetus, hepatic output of glutamate may substitute for hepatic glucose output and limit amino acid oxidation since fetal hepatic oxygen consumption is relatively fixed (27, 28). Moreover, there are shifts in these nutrient shuttles between the placenta and the FGR fetus (15) that may affect substrate availability for HGP.

The objective of this study was to determine the carbon substrates and associated pathways used by the FGR liver to support increased HGP. We hypothesized that FGR fetuses would have increased hepatic utilization of lactate and glucogenic amino acids, at the expense of oxidation, to support early and persistent HGP. To test this, we used fetal hepatic catheterization to directly measure in vivo hepatic metabolism in normal and FGR sheep fetuses coupled with ^13^C-glucose tracing, hepatic tissue–based molecular and biochemical analyses, and mechanistic studies in primary fetal hepatocytes. These results provide what we believe are new insights regarding early metabolic effects in the liver of the FGR fetus.

## Results

### Decreased hepatic blood flow and increased HGP with FGR.

**Figure 1:**
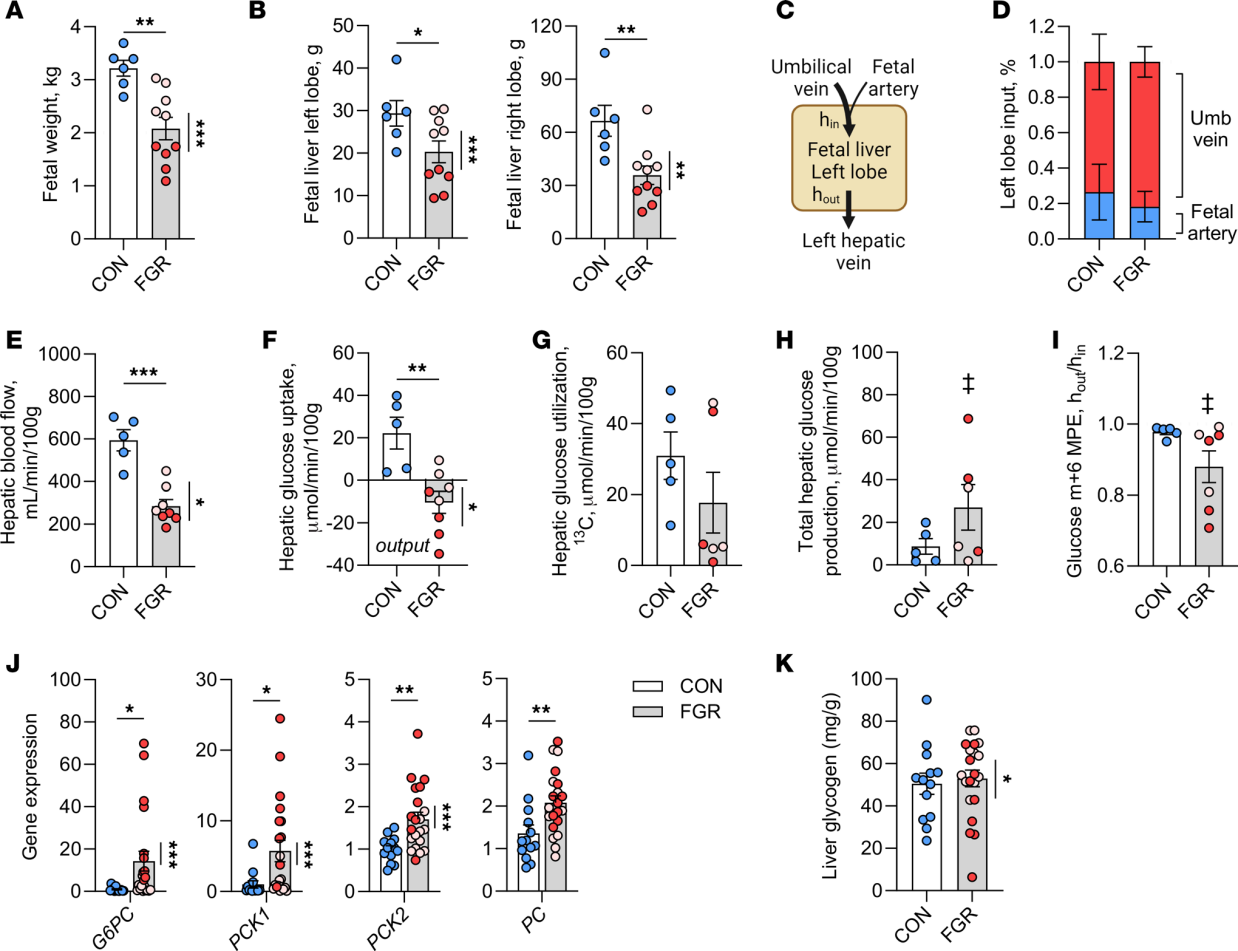
Decreased hepatic blood flow and increased hepatic glucose production in FGR fetuses. (**A**) Fetal weight in CON and FGR groups. Dashed line at 2 kg indicates the separation between moderate (light pink) and severe (dark red) FGR. (**B**) Fetal left and right lobe liver weights. (**C**) Left hepatic catheterization method showing input (h_in_) and output (h_out_) across the left lobe. (**D**) Percentage contribution of umbilical vein and fetal artery blood supply to the left lobe. (**E**) Fetal hepatic blood flow normalized per 100 g of liver weight. (**F**) Net hepatic glucose uptake. (**G**) Hepatic glucose utilization. (**H**) Total hepatic glucose production (sum of utilization and net uptake). (**I**) Glucose tracer enrichment (m+6 MPE) ratio across the fetal liver. (**J**) Expression of gluconeogenic genes in liver tissue. (**K**) Hepatic tissue glycogen content. Means ± SEM are shown (*n* = 5–6 CON, 10 FGR in panels **A**–**I** and *n* = 13 CON, 21 FGR in panels **J** and **K**). Results comparing CON (white bar) versus FGR (gray bar) groups were analyzed by 2-tailed Student’s *t* test, except panel **I**, where a Welch’s *t* test was used. In panels **H** and **I**, a 1-sample Wilcoxon test was used to test whether each group was greater than zero (panel **H**) or less than 1.0 (panel **I**) and differences are indicated with a double dagger. Weight-threshold differences comparing moderate versus severe FGR were analyzed by 2-tailed Student’s *t* test and are indicated by a vertical line. ‡*P* < 0.05; *P < 0.05; **P < 0.01, ***P < 0.001.

In our sheep model of FGR, There was a range in the severity of FGR (Figure 1A), with some fetuses weighing less than 2 kg (dark red circles) and others weighing more than 2 kg (light pink circles). All FGR fetuses were analyzed together and compared to normal (CON) fetuses. Secondary analyses were performed to test for weight-threshold differences within the FGR group. Liver left lobe and right lobe weights were 30% and 45% lower, respectively, in FGR compared with CON fetuses, with a significant weight-threshold effect in the FGR group (Figure 1B). Total liver weight, representing the sum of the left and right lobes, when normalized to fetal weight was similar between groups (CON: 2.95%; FGR: 2.63%).

Direct catheterization was used to measure nutrient and oxygen concentrations entering (h_in_) and leaving (h_out_) the left lobe of the fetal liver (Figure 1C) coupled with hepatic blood flow measurements (3) and application of the Fick principle to calculate in vivo net hepatic nutrient and oxygen uptake rates. In CON and FGR fetuses, over 80% of the blood supply to the left lobe of the fetal liver was from the umbilical vein, with the remainder supplied by fetal artery blood (Figure 1D). In FGR fetuses, hepatic blood flow across the left lobe was 65% lower on an absolute basis (CON: 176.6 mL/min; FGR: 61.9 mL/min; *P* < 0.001). Weight-normalized hepatic blood flow rates were 52% lower in FGR compared with CON fetuses, with a significant weight-threshold effect in the FGR group (Figure 1E). All subsequent flow-based rates were normalized per 100 g of liver weight.

FGR fetuses had lower arterial plasma glucose concentrations (Table 1) and increased net hepatic glucose output (negative uptake, Figure 1F) compared with CON fetuses, all of which had net hepatic glucose uptake. There was no difference in hepatic glucose utilization rates (Figure 1G). Total HGP, measured as the sum of glucose output and utilization, in the FGR group was greater than zero (Figure 1H), whereas in the CON group, this value was not greater than zero and net uptake was equal to utilization. Furthermore, there was glucose tracer dilution (^13^C-glucose m+6 ratio h_out_/h_in_) across the left lobe of the FGR fetal liver, supporting HGP (Figure 1I). Expression of the major gluconeogenic genes, *G6PC*, *PCK1*, *PCK2*, and *PC*, was increased in FGR compared with CON fetal liver tissue (Figure 1J), with no difference in hepatic glycogen content (Figure 1K). Fetuses with severe versus moderate FGR had greater hepatic glucose output, higher expression of *G6PC*, *PCK1*, and *PCK2*, and lower hepatic glycogen content. In addition, FGR fetuses had an endocrine milieu favoring glucose production, with lower concentrations of insulin and higher counterregulatory hormones (cortisol, glucagon, and norepinephrine) (Table 1).

### Lower hepatic oxidative metabolism in FGR fetus.

**Figure 2:**
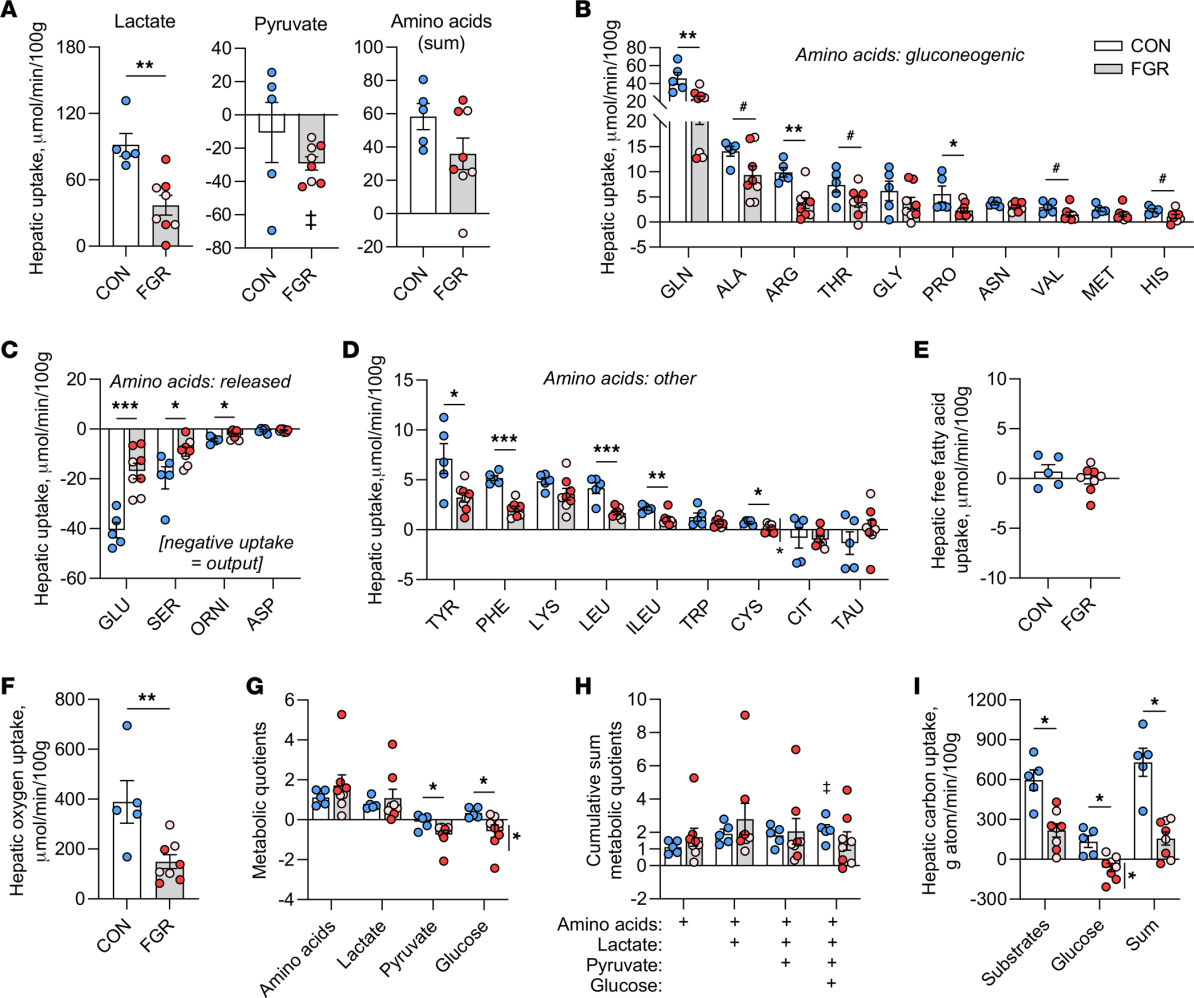
Lower hepatic uptake of carbon substrates and oxygen consumption in FGR fetus. (**A**) Net hepatic lactate, pyruvate, and sum of all individual amino acid uptake rates measured in CON and FGR fetuses. Individual amino acids uptake rates are grouped as those that are (**B**) considered gluconeogenic, (**C**) normally released by the fetal liver, and (**D**) all other amino acids. (**E**) Net hepatic free fatty acid uptake. (**F**) Hepatic oxygen consumption rates. (**G**) Hepatic nutrient/oxygen metabolic quotients. (**H**) Cumulative sums of metabolic quotients. (**I**) The net carbon uptake of substrates (defined as sum of lactate, pyruvate, and all amino acids), glucose, and the sum of substrates plus glucose. (**I**) Means ± SEM are shown (*n* = 5 CON, 10 FGR). Results comparing CON (white bar) versus FGR (gray bar) groups were analyzed by 2-tailed Student’s *t* test. Weight-threshold differences comparing moderate (light pink) versus severe (dark red) FGR were analyzed by 2-tailed Student’s *t* test and are indicated by a vertical line. *P < 0.05; **P < 0.01, ***P < 0.001, ^#^*P* < 0.15.

We next measured the net hepatic uptake of substrates that may provide carbons for HGP in the FGR fetus. Lactate and pyruvate are major 3-carbon carbohydrate precursors for gluconeogenesis in adults. Arterial plasma lactate and whole blood pyruvate concentrations were 80% and 50% higher, respectively, in FGR compared with CON fetuses, with higher concentrations in severe compared with moderate FGR fetuses (Table 1). However, hepatic lactate uptake was reduced by 60% in FGR compared with CON fetuses (Figure 2A). Furthermore, FGR fetuses had net output of pyruvate (mean greater than zero, *P* < 0.05; Figure 2A), whereas in the CON group net hepatic pyruvate uptake/output was not greater than zero. Amino acids fuel approximately 40% of oxidative metabolism in the normal fetal liver and many are gluconeogenic, with a net contribution toward glucose production (3, 29). FGR fetuses had lower serine, asparagine, and isoleucine arterial plasma amino acid concentrations (Table 1). Total hepatic amino acid uptake was not different in FGR compared to CON fetuses (Figure 2A). However, the uptake of many gluconeogenic amino acids was reduced. This included decreased glutamine, arginine, and proline (Figure 2B), with lower (*P* < 0.1) alanine, threonine, valine, and histidine uptake. The net hepatic output of glutamate, serine, and ornithine was lower (Figure 2C). The net hepatic uptake of tyrosine, phenylalanine, isoleucine, leucine, and cysteine also was lower (Figure 2D). Of all the amino acids, only cysteine uptake was lower in fetuses with severe compared with moderate FGR. Free fatty acids via their oxidation may also provide carbons or ATP for HGP (30). FGR fetuses, however, had no difference in free fatty acid concentrations compared to CON fetuses (Table 1) and no detectable hepatic uptake (Figure 2E). These data suggest mechanisms other than increased hepatic uptake of lactate, pyruvate, amino acids, or fatty acids are supporting increased HGP in FGR fetuses.

We next determined the effects on hepatic oxygen consumption and oxidative metabolism. FGR fetuses were hypoxemic, with lower arterial blood oxygenation (Table 1). Fetuses with severe compared with moderate FGR were more hypoxemic. The hepatic oxygen consumption rate was 62% lower in FGR compared with CON fetuses (Figure 2F). Metabolic quotients are a flow-independent assessment of substrate relative to oxygen uptake on a molar basis (31). The hepatic metabolic quotients for the sum of all amino acids and lactate were similar between CON and FGR fetuses, while the quotients for glucose and pyruvate were lower and negative (reflecting net output) in FGR fetuses (Figure 2G). The cumulative sum of the metabolic quotients starting with amino acids and adding lactate exceeded the threshold needed to sustain oxidative metabolism (i.e., >1.0) by nearly 2-fold in both CON and FGR groups (Figure 2H). Given the small relative decrease and increase by pyruvate and glucose, respectively, the sum of quotients exceeded 1.0 in the CON liver. In contrast, in the FGR liver, after accounting for the output of glucose and pyruvate, the sum of the metabolic quotients was not greater than 1.0 (Figure 2H). Moreover, the sum on a per-carbon-atom basis of the net hepatic uptake of all amino acids, lactate, and pyruvate was 60% lower in FGR compared with CON fetuses (Figure 2I). The sum of carbon uptake of these substrates plus the respective net flux of glucose was 80% lower in FGR compared with CON fetuses (Figure 2I). The hepatic metabolic quotient and carbon uptake for glucose were both lower in severe compared with moderate FGR fetuses. Together, lower hepatic uptake of carbon substrates and oxygen consumption demonstrate lower hepatic oxidative metabolism in the FGR fetus.

### Identification of metabolic pathways supporting HGP.

**Figure 3:**
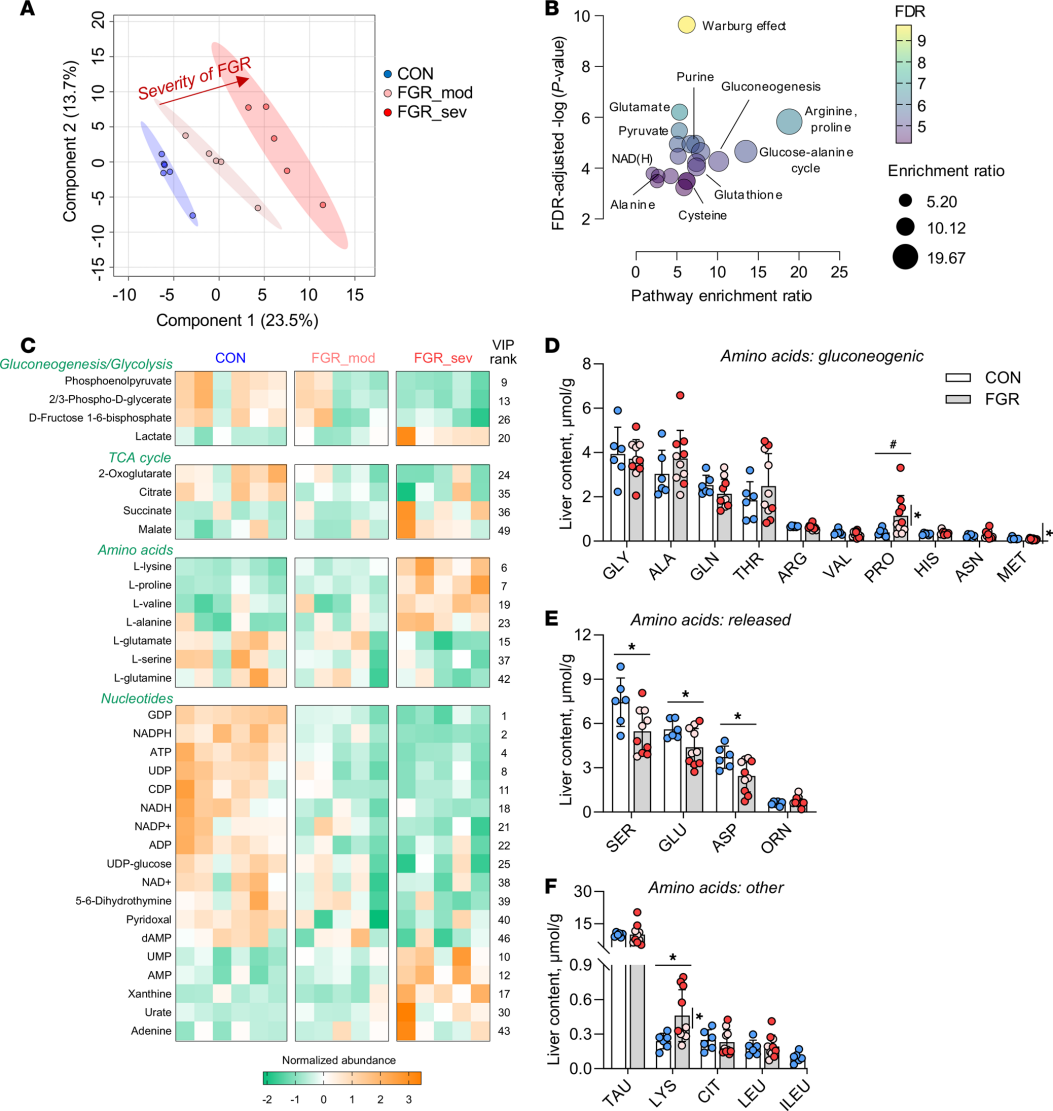
Hepatic tissue metabolites and amino acids. Targeted metabolomic profiling with relative quantification in CON and FGR liver samples. (**A**) PLS-DA plot showing separation between groups. (**B**) Pathway enrichment analysis using the top 50 metabolites with the highest VIP scores and plotting enrichment ratios and FDR-adjusted significance (*P* < 0.00007). Pathways of interest are labeled. (**C**) Heatmap showing relative normalized abundance of metabolites within the major pathways. VIP rank from component 1 is shown. The hepatic tissue content of individual amino acids is shown for amino acids that are (**D**) gluconeogenic, (**E**) normally released by the fetal liver, and (**F**) all other amino acids. Means ± SEM are shown (*n* = 5–6 CON, 10 FGR). Results comparing CON (white bar) versus FGR (gray bar) groups were analyzed by 2-tailed Student’s *t* test. Weight-threshold differences comparing moderate (light pink) versus severe (dark red) FGR were analyzed by 2-tailed Student’s *t* test and are indicated by a vertical line. *P < 0.05; ^#^*P* < 0.15.

In the setting of lower substrate uptake by the FGR liver, we used targeted metabolomics profiling with relative quantification to identify intrahepatic pathways that may direct carbon substrates for HGP. Multivariate partial least squares discriminant analysis (PLS-DA) of the 152 metabolites detected in fetal liver tissue showed distinct separation between CON and FGR groups, including within the FGR group based on severity of growth restriction (Figure 3A). The 50 metabolites with the highest variable in projection (VIP) scores were subjected to pathway enrichment analysis (Supplemental Tables 1 and 2; supplemental material available online with this article; https://doi.org/10.1172/jci.insight.176497DS1). This identified pathways of interest associated with the Warburg effect, glucose-alanine cycle, gluconeogenesis, pyruvate metabolism, purine metabolism, NAD metabolism, and glutathione metabolism, in addition to other pathways associated with amino acid metabolism (Figure 3B). Metabolites represented in these pathways belong to broad categories related to gluconeogenesis/glycolysis, TCA cycle, amino acids, and nucleotides, as shown by heatmap with relative abundance by samples within CON and FGR groups (Figure 3C). We confirmed these relative differences in amino acid abundance using absolute quantification. Total hepatic tissue amino acid content was not different between groups (Figure 3D). The hepatic content of serine, glutamate, and aspartate was decreased in the FGR liver (Figure 3E), matching the lower hepatic release of these amino acids (see Figure 2C). The hepatic content of lysine was increased, and proline trended higher (Figure 3F). Within the FGR group, fetuses with severe FGR had higher proline and lysine and lower methionine content. These results suggest that metabolic responses associated with Warburg-like shifts in metabolism may direct available carbons toward HGP in the FGR liver.

### Decreased hepatic pyruvate oxidation and increased lactate production.

**Figure 4:**
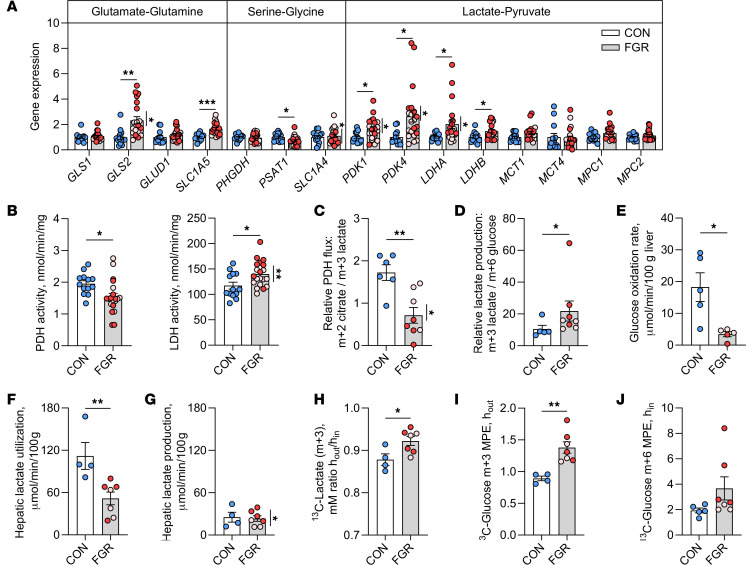
Amino acid, pyruvate, and lactate metabolism. (**A**) Gene expression in CON and FGR liver tissue. (**B**) Hepatic PDH and LDH activity. ^13^C-labeling of metabolites in liver tissue representing (**C**) PDH flux (ratio of m+2 citrate relative to m+3 lactate) and (**D**) lactate production (m+3 lactate relative to m+6 glucose). (**E**) Hepatic (in vivo) glucose oxidation rate. (**F**) Hepatic lactate utilization rate. (**G**) Endogenous hepatic lactate production rate (difference between lactate utilization in panel **F** and net hepatic uptake in Figure 2A). (**H**) Ratio of ^13^C-lactate (m+3) measured across the fetal hepatic circulation (h_out_/h_in_). (**I**) Glucose m+3 enrichment (MPE) measured in hepatic output (h_out_). (**J**) Plasma glucose m+6 enrichment (MPE) measured in hepatic input (h_in_). Means ± SEM are shown (*n* = 13 CON, 21 FGR in panels **A** and **B** and *n* = 5 CON, 7–10 FGR in panels **C**–**H**). Results comparing CON (white bar) versus FGR (gray bar) groups were analyzed by 2-tailed Student’s *t* test. Weight-threshold differences comparing moderate (light pink) versus severe (dark red) FGR were analyzed by 2-tailed Student’s *t* test and are indicated by a vertical line. *P < 0.05; **P < 0.01, ***P < 0.001.

We validated targets involved in Warburg-like metabolism to determine whether the FGR liver, like a cancer cell, employs similar mechanisms to support proliferation and growth (32–34). Given our metabolite results, we focused on pyruvate oxidation, lactate production, and amino acid metabolism. FGR fetuses had increased hepatic tissue expression of *SLC1A5*, a glutamine uptake transporter, and *GLS2*, which converts glutamine to glutamate (Figure 4A). Expression of the serine biosynthesis regulator, *PSAT1*, was also decreased. Expression of the pyruvate dehydrogenase (PDH) kinases, *PDK1* and *PDK4* (Figure 4A), which phosphorylate and inhibit the activity of PDH, was increased. Expression of lactate dehydrogenase genes, *LDHA* and *LDHB*, was also increased. Expression of the monocarboxylate transporter *MCT1* and mitochondrial pyruvate transporter *MPC1* were also higher (*P* < 0.10). In support of increased lactate production and decreased pyruvate oxidation in the FGR liver, hepatic tissue enzymatic activity of LDH was increased and that of PDH was decreased (Figure 4B). Fetuses with severe compared with moderate FGR had higher expression of *GLS2*, *SLC1A4*, *PDK1*, *PDK4*, and *LDHA* and higher LDH activity.

Next, we measured ^13^C-metabolite labeling in liver tissue (Supplemental Figure 1A) and in vivo hepatic rates of glucose oxidation and lactate production. In support of decreased PDH activity, the hepatic tissue abundance of m+2 citrate relative to m+3 lactate was decreased by 60%, with lower PDH flux in severe versus moderate FGR (Figure 4C). The hepatic tissue abundance of m+4 citrate relative to m+3 malate and abundance of m+5 citrate relative to m+3 malate, other measures of PDH flux, were not different between CON and FGR groups; however, within the FGR group, both ratios were lower in fetuses with severe versus moderate FGR (Supplemental Figure 1B). The hepatic tissue abundance of m+3 lactate relative to m+6 glucose was increased by more than 2-fold, supporting increased lactate production (Figure 4D). FGR fetuses also had an 80% reduction in hepatic glucose oxidation rate (Figure 4E). Hepatic lactate utilization rates were nearly 2-fold lower in FGR compared with CON fetuses (Figure 4F). These rates exceeded net uptake rates (shown in Figure 2A), demonstrating endogenous hepatic lactate production rates between groups (Figure 4G). While the rate of lactate production was not different, it did represent nearly 50% of total lactate utilization (Supplemental Figure 1C). Furthermore, the ratio of labeled hepatic lactate output relative to uptake (h_out_/h_in_) was 15% higher in FGR compared with CON fetuses (Figure 4H). In support of intrahepatic utilization of 3-carbon intermediates (i.e., lactate and pyruvate), the m+3 enrichment of plasma glucose leaving the hepatic circulation was 50% in the FGR fetus (Figure 4I) and there was no difference in the precursor glucose m+6 tracer enrichment (Figure 4J). These data suggest higher intrahepatic lactate production and incorporation into newly synthesized glucose.

### Persistently higher glucose production and preference for lactate in isolated primary hepatocytes from FGR fetus.

**Figure 5:**
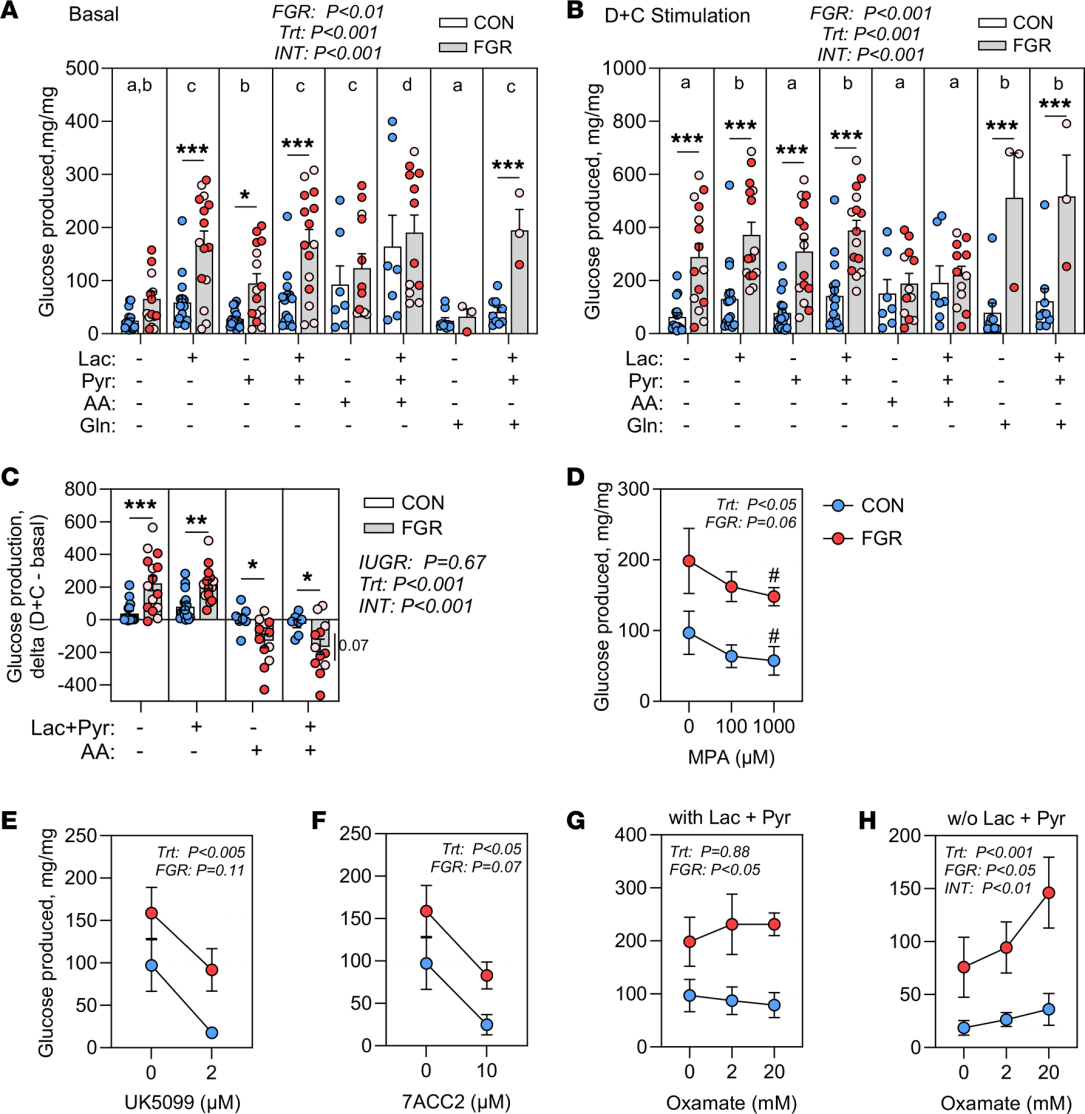
Glucose production in isolated fetal hepatocytes. Glucose production measured in isolated primary hepatocytes from CON (*n* = 16) and FGR (*n* = 15) fetuses under (**A**) basal or (**B**) stimulated conditions with dexamethasone and cAMP (D+C). Substrates were provided: 20 mM lactate (Lac), 2 mM pyruvate (Pyr), 20% TrophAmine (w/vol, amino acids, AA), or 2 mM glutamine (Gln). (**C**) The effect of stimulation with D+C represented as the increase with D+C compared to basal without substrates (none) or with Lac+Pyr or AA. (**D**) Effect of 3-mercaptopicolinic acid (MPA) on glucose production in CON (*n* = 3) and FGR (*n* = 2) hepatocytes. Effects of UK5099 (**E**), 7ACC2 (**F**), and oxamate (**G** and **H**) in CON (*n* = 3) and FGR (*n* = 4) hepatocytes. Experiments in **D**–**G** were performed with hormone stimulation (D+C) and 20 mM lactate and 2 mM pyruvate. In **H**, D+C stimulation without lactate and pyruvate was used. Experiments were analyzed with 2-way ANOVA. ANOVA main and interaction effects are shown. In panels **A** and **B**, different letters represent differences (*P* < 0.05) among substrate combinations by posttest comparison with Students *t* test. ^#^*P* < 0.10; *P < 0.05; **P < 0.01, ***P < 0.001 represent posttest comparisons. Weight threshold differences comparing moderate (light pink) versus severe (dark red) FGR were analyzed by 2-tailed Student’s *t* test and are indicated by a vertical line.

We next isolated primary hepatocytes from CON and FGR fetuses to test for persistently increased glucose production rates and substrate preference. Substrate preference for glucose production from pyruvate, lactate, or amino acids was measured in glucose-free media under basal (Figure 5A) and stimulated conditions with dexamethasone and cAMP (D+C; Figure 5B) to model the higher counterregulatory hormone milieu in FGR fetuses (Table 1). Across all conditions, hepatocytes from FGR fetuses had increased glucose production (FGR main effect *P* < 0.001). Lactate and lactate plus pyruvate, but not pyruvate alone, increased glucose production compared with the condition without substrates under both basal and D+C stimulation. A mixture of essential and nonessential amino acids lacking glutamine increased glucose production in both CON and FGR hepatocytes under basal conditions, yet had no added effects when combined with D+C stimulation. Glutamine alone increased stimulated but not basal glucose production. Within substrate treatment groups, FGR hepatocytes had higher glucose production compared with CON hepatocytes in the presence of lactate, pyruvate, or the combination of both under basal and stimulated conditions (Figure 5, A and B). Glucose production was also augmented with the combination of glutamine, pyruvate, and lactate only in FGR hepatocytes. There were no fetal weight-threshold effects within the FGR group. The increase in glucose production with D+C stimulation was greater in FGR compared with CON hepatocytes in the absence of substrates (Figure 5C), which could reflect glycogenolysis. The combination of lactate plus pyruvate also augmented D+C-stimulated glucose production in FGR compared with CON hepatocytes (Figure 5C). Unexpectedly, amino acids impaired D+C-stimulated glucose production in FGR but not CON hepatocytes, with greater effect in hepatocytes from fetuses with severe FGR (Figure 5C). Together, these results support cell-intrinsic effects in FGR compared with CON hepatocytes that enable greater glucose production, a preference for lactate and pyruvate as substrates, and increased response to D+C stimulation.

In a second cohort of isolated primary hepatocytes from CON and FGR fetuses, we next sought to test (a) whether increased glucogenic capacity in FGR hepatocytes was dependent on PEPCK-C and (b) the specificity for lactate and pyruvate as substrates (Supplemental Figure 2A). FGR hepatocytes in this cohort had higher glucose production in response to lactate plus pyruvate and D+C (Supplemental Figure 2B), like the larger cohort (Figure 5, A and B). In the following experiments, the condition with maximally stimulated glucose production in the presence of D+C and lactate plus pyruvate was used. The PEPCK inhibitor, 3-mercaptopicolinic acid (MPA), decreased glucose production by 40% and 25%, respectively, in CON and FGR hepatocytes (Figure 5D). To test the specificity for lactate and pyruvate, we used inhibitors of the monocarboxylate transporters to inhibit the uptake of pyruvate and lactate. Both UK5099 and 7ACC2 decreased glucose production, with 80% and 75% suppression in CON hepatocytes and 42% and 48% suppression in FGR hepatocytes (Figure 5, E and F). We also tested the effect of inhibiting LDH and endogenous lactate production with oxamate. In the presence of exogenous lactate and pyruvate, oxamate had little effect (Figure 5G). In the absence of exogenous lactate and pyruvate, oxamate increased glucose production by 2-fold in both CON and FGR hepatocytes (Figure 5H). Thus, when stimulated to produce glucose, both CON and FGR hepatocytes use lactate and pyruvate for gluconeogenesis.

### Activation of stress signaling and redox regulation.

**Figure 6:**
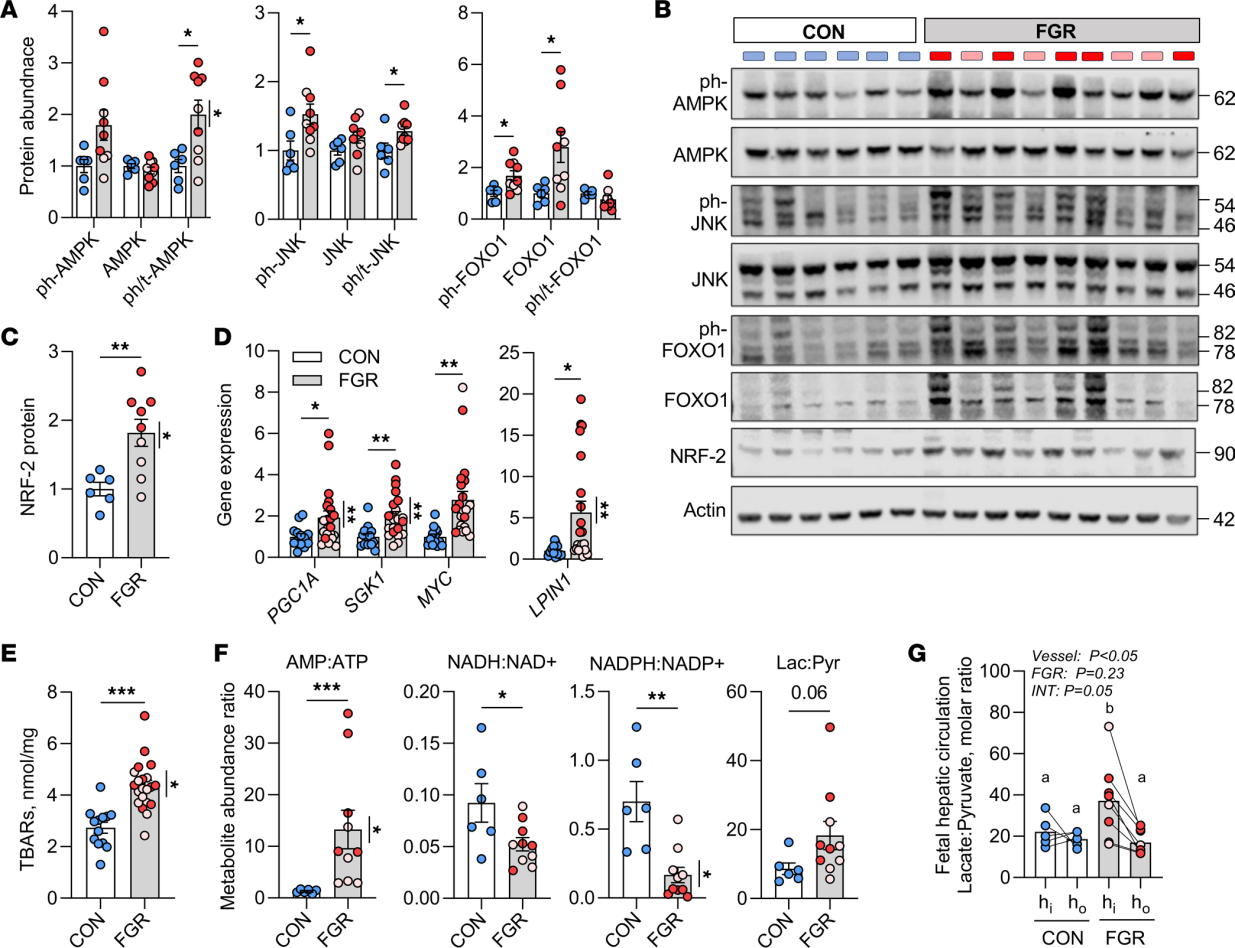
Nutrient signaling and oxidative stress. (**A**) Protein expression in CON and FGR livers for phosphorylated (ph-) and total protein abundance of AMPK (T172), JNK (T183/Y185), and FOXO1 (T24). Ratios of phosphorylated to total protein were calculated. (**B**) Representative Western blot images, with molecular weights of protein bands indicated. Actin shown for equality of loading. (**C**) Total abundance of NRF-2. (**D**) Hepatic expression of genes associated with nutrient and stress signaling. (**E**) Hepatic TBARS content. (**F**) Liver tissue metabolite abundance ratios for AMP/ATP, NADH/NAD+, NADPH/NADP+, and lactate/pyruvate (Lac:Pyr). (**G**) Blood lactate/pyruvate ratio measured across the fetal hepatic circulation with input (h_in_) and output (h_out_) shown. Means ± SEM are shown (*n* = 6 CON, 9–10 FGR, except panel **E** with *n* = 14 CON, 21 FGR). Results were analyzed by Student’s *t* test. *P < 0.05, **P < 0.01, ***P < 0.001. When *P* > 0.05, the value is shown. Data points on graphs or blot lanes representing samples from FGR fetuses with moderate (pink) or severe (red) growth restriction are indicated. Weight-threshold differences comparing moderate (light pink) versus severe (dark red) FGR were analyzed by 2-tailed Student’s *t* test and are indicated by a vertical line.

We next sought to determine whether reduced hepatic oxidative metabolism activates stress pathways in the FGR liver. Phosphorylation of the energy sensor AMPK was 2-fold higher in the FGR liver (Figure 6, A and B). Consistent with our prior work (4), phosphorylation of the stress kinase JNK and FOXO1, a regulatory node between stress signaling and insulin action, was higher in the FGR liver (Figure 6, A and B). Protein abundance of the oxidative stress protein NRF-2 was nearly 2-fold higher (Figure 6, B and C). Expression of *PGC1A*, *SGK1*, *MYC*, and *LPIN1* genes, all stress-sensitive targets (20), were increased in FGR liver (Figure 6D). Hepatic tissue thiobarbituric acid–reactive substances (TBARS) content, a marker of oxidative stress, was 60% higher in FGR livers (Figure 6E). FGR livers also had a higher AMP/ATP ratio, consistent with AMPK activation, and lower ratios of NADH/NAD+ and NADPH/NADP+, supporting a lower tissue redox state (Figure 6F). Despite higher lactate tissue abundance (see Figure 3C), the hepatic tissue lactate/pyruvate ratio only tended to be higher (*P* = 0.06, Figure 6F). In the FGR group, weight-threshold effects were found for AMPK activation, NRF-2 abundance, expression of *PGC1A*, *SGK1*, and *LPIN1*, TBARS content, AMP/ATP, and NADPH/NADP+. Interestingly, the blood lactate/pyruvate concentration ratio entering the fetal liver (h_in_) was nearly 2-fold higher in the FGR fetus, whereas this ratio leaving the liver (h_out_) was lower and similar to the ratio across the CON fetal liver (Figure 6G). Thus, lower hepatic oxidative metabolism produces nutrient and energetic stress in the FGR liver, with increased hepatic pyruvate output serving to improve intrahepatic and systemic redox balance.

## Discussion

**Figure 7:**
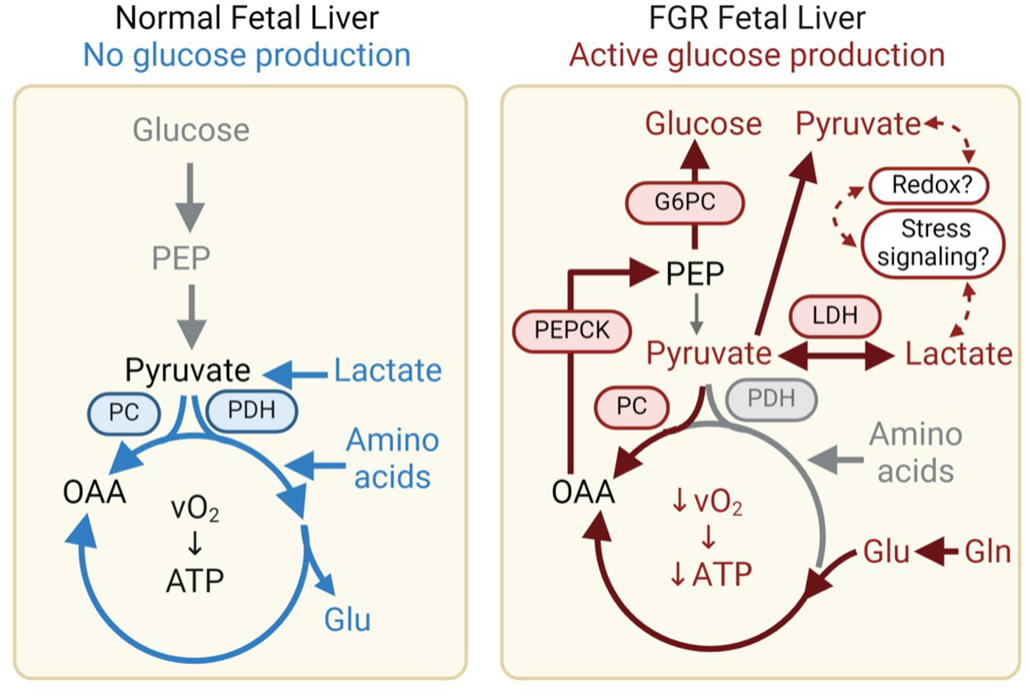
Increased hepatic glucose production and reduced hepatic oxidative metabolism in FGR fetus. In the normal fetus, hepatic oxidative metabolism is fueled by lactate and amino acids, with a small net uptake of glucose. Herein, we demonstrate that the FGR fetus has increased HGP and net glucose output, with lower oxidative metabolism. Our data support increased lactate production, decreased pyruvate oxidation, and decreased glutamate output as putative mechanisms that make carbons available for glucose production. The FGR liver also has evidence of nutrient and energetic stress and redox imbalance. This may be a consequence of reduced oxidative metabolism and offset by increased pyruvate output.

FGR fetuses had an early activation of HGP and reduced hepatic oxidative metabolism, which may make carbons available for HGP, but also produce nutrient and energetic stress (Figure 7). Our study is the first to our knowledge to demonstrate in vivo net hepatic glucose output measured directly in FGR fetuses, in contrast with net hepatic glucose uptake in CON fetuses. We also demonstrate that FGR fetuses had increased intrahepatic lactate production, inhibition of pyruvate oxidation, increased pyruvate output, and decreased glutamate output. Furthermore, FGR fetuses had lower hepatic oxidative metabolism, with lower ATP production and generation of NADH-reducing equivalents, suggesting an energetic debt. Indeed, the FGR liver had activation of energetic and oxidative stress pathways. These effects were more pronounced in severe compared with moderate FGR fetuses. These results advance our understanding of the availability of carbon substrates and energy cofactors that the FGR liver may use in the setting of hypoglycemia, hypoxemia, and energetic debt to support HGP, at the expense of oxidative metabolism.

FGR fetuses had increased hepatic glucose output, confirming that the liver is a major site of endogenous glucose production (4). In support of active gluconeogenesis, the FGR liver had an upregulation of the genes (*PCK1*, *PCK2*, *PC*) that regulate the use of 3-carbon intermediates for new glucose synthesis. Furthermore, inhibition of PEPCK-C with 3-MPA decreased glucose production in isolated FGR hepatocytes. Glycogenolysis may also be active in the FGR liver, especially in fetuses with severe FGR, which had lower hepatic tissue glycogen content. Moreover, FGR hepatocytes produced glucose in the absence of substrates, albeit at lower rates compared with when substrates were provided, supporting the utilization of endogenous substrates to synthesize glucose or the breakdown of stored glycogen. Our results confirm studies in newborn and postnatal rodent models of FGR demonstrating increased HGP and dysregulation of *PCK1* (10, 11) and extend these findings by demonstrating these effects in the fetus (at 0.9 gestation length) before birth. In human cohorts, children and adults born with FGR have increased T2D (35–39); however, many studies use glucose tolerance testing for diagnosis, which cannot identify liver-specific effects. Studies that have measured HGP and hepatic insulin sensitivity in lean adult men born with FGR report impaired insulin suppression of HGP (40–43). Together, these studies support the developmental programming of dysregulated HGP in both animal models and humans.

We hypothesized that the FGR fetus would have increased net hepatic uptake of lactate and glucogenic amino acids, which would provide carbons for increased HGP. In contrast with our hypothesis, the hepatic uptake of lactate and gluconeogenic amino acids, including glutamine and alanine, was decreased and instead, hepatic output of glutamate and serine was decreased. In addition, the hepatic tissue content of glutamate, serine, and aspartate was decreased, yet lactate content was increased. Using metabolite profiling to identify other mechanisms that may direct carbons for HGP, we found an upregulation of Warburg-like metabolism. In support, the expression of genes regulating glutamine utilization and lactate-pyruvate metabolism was increased (18) and enzymatic activity and ^13^C-labeling data demonstrate decreased PDH activity and pyruvate oxidation and increased LDH activity and lactate production. Moreover, isolated hepatocytes from FGR compared with CON fetuses had greater intrinsic capacity for glucose production, with a preference for lactate and pyruvate as substrates that was blocked when monocarboxylate (i.e., pyruvate and lactate) transport was inhibited with 7ACC2 or UK5099 treatment. In addition, the plasma ^13^C-glucose m+3 enrichment leaving the FGR liver was higher, supporting increased gluconeogenesis from ^13^C-labeled 3-carbon precursors (i.e., lactate, pyruvate, alanine), produced following the intrahepatic or extrahepatic metabolism of the U-^13^C-glucose (m+6) tracer (see Supplemental Figure 1). Previously, we have shown that amino acids stimulate glucose production in normal fetal hepatocytes (44) and that fetal hepatic amino acid uptake is greater during hypoglycemia when HGP is active (3). Herein, the provision of a mixed amino acid solution stimulated glucose production in both CON and FGR hepatocytes, while lactate stimulated glucose production only in FGR hepatocytes. This suggests that pathways enabling the utilization of amino acids for glucose production are active in isolated hepatocytes from both CON and FGR fetuses, while only FGR hepatocytes have cell-intrinsic effects supporting lactate utilization. Alternatively, the in vitro study conditions, including a relatively high oxygen supply compared with the in utero environment, may produce in vitro effects that are different from those operating in utero. This may explain why hepatic amino acid uptake is increased in fetal sheep with hypoglycemia (3) but not FGR, despite both having active HGP. Interestingly, lower hepatic glutamate output when HGP is active provides compelling support for the hypothesis that glutamate output substitutes for glucose output in the normal fetal liver (28). Overall, these results identify a cell-intrinsic effect in FGR hepatocytes for increased glucose production and a putative preference for lactate as a substrate.

Hepatic pyruvate output was unexpected in FGR fetuses because pyruvate is a major gluconeogenic substrate. We speculate that the pool of 3-carbon intermediates exceeds what can be used for gluconeogenesis or oxidation, due to decreased PDH activity, and excess is released as pyruvate. Interestingly, emerging studies demonstrate that intracellular lactate shuttling (45, 46) and systemic lactate and pyruvate concentrations and their ratio may function as a circulating redox buffer (47). Thus, we speculate that increased cytosolic lactate production from pyruvate may be offset by the mitochondrial oxidation of lactate back to pyruvate and used for gluconeogenesis; however, additional studies are needed to test this and assess the redox consequences of such shuttling. In addition, the blood lactate/pyruvate ratio was higher entering compared with leaving the liver in the FGR compared with the CON fetus, suggesting that pyruvate output by the FGR liver lowers the lactate/pyruvate ratio and may restore systemic redox balance. Pyruvate produced from the fetal liver may also be shuttled to the placenta as a mechanism to allow maternal glucose to be spared from oxidation in the placenta and transported to the fetus or converted to lactate by the placenta, while pyruvate produced from the fetus is oxidized by the placenta, as we have shown in FGR and hypoxemic pregnancies (15, 48).

Our results identify nutrient and stress signaling pathway activation as a potential mechanism that coordinates the metabolic responses that make carbon and energy substrates available for HGP in the FGR liver (4, 20). Consistent with our prior studies, FGR livers had increased JNK and FOXO1 signaling and activation of stress-sensitive genes (4, 20). Extending these results, FGR livers had evidence of increased nutrient and energetic stress, with higher AMPK activation, NRF-2 abundance, TBARS, and lactate/pyruvate ratio and lower NAD(P)H/NAD(P)+ ratios. Hepatic urate concentrations were also increased. Prior studies have shown higher urate plasma concentrations in FGR and hypoxemic fetuses (22, 49), which may result from increased purine catabolism and oxidative stress. Moreover, the FGR liver demonstrates features of Warburg-like metabolism (32–34), including an upregulation of *MYC* and *PGC1A*, lower pyruvate oxidation, increased lactate production, and shifts in amino acid metabolism. This suggests that like cancer cells, FGR liver cells have developed strategies to divert carbon substrates into pathways for growth, oxidation, or glucose production. Given the interaction observed in vitro in isolated FGR hepatocytes with D+C and amino acids, we speculate that stress hormone signaling may antagonize amino acid utilization for HGP and divert these carbon skeletons for oxidation or growth.

Reduced hepatic oxygen consumption and oxidative metabolism in the FGR fetus were paralleled by lower total net hepatic substrate uptake, ATP, and NAD(P)H-reducing equivalents. This may produce a lower ATP/ADP ratio and a more positive free energy value for ATP hydrolysis (ΔG-ATP) in the FGR liver. Since gluconeogenesis is an endergonic reaction, this may enable lactate-fueled HGP to be more thermodynamically favorable. However, the energetic cost of making glucose likely compromises other anabolic pathways, including fetal growth. Indeed, the sum of nutrient/oxygen metabolic quotients was not greater than 1.0. This demonstrates a matching between the hepatic uptake of carbon substrates to fuel oxidative metabolism and support glucose and pyruvate output, leaving limited substrates available for growth. FGR fetuses also have lower insulin and IGF-1 concentrations (15, 50), yet no differences in hepatic mTOR signaling (20). We speculate that lower anabolic growth signals, independent of mTOR signaling, together with an upregulation of stress signaling pathways and lower nutrient supply limit hepatic oxidative metabolism and growth. Interestingly, fetal liver weight normalized to fetal body weight was not different in FGR compared to CON fetuses, yet the proportion of whole-body oxygen consumption used by the fetal liver was reduced from 35% in CON to 14% in FGR fetuses (calculated from Figure 2E) (15), supporting the relative restriction of hepatic oxidative metabolism, but not growth. This contrasts with skeletal muscle, which has reduced weight-specific oxidative metabolism and growth (50), and highlights the importance of tissue-specific differences that contribute to the hypometabolic phenotype in FGR fetuses (16, 21).

The FGR fetus is hypoglycemic, hypoxemic, and has an endocrine milieu with decreased insulin and increased counterregulatory hormones, all of which may contribute to increased HGP. Our prior studies demonstrate that hypoglycemia alone is sufficient to increase HGP in the late-gestation fetus (3) and that hypoxemia alone potentiates gluconeogenic gene activation, but does activate HGP, likely because glucose concentrations are not decreased (51). Herein, isolated hepatocytes from FGR fetuses have increased glucose production rates even when studied under ambient oxygen conditions, with relatively more hyperoxia compared with the hypoxia present in utero. Our results also demonstrate that the severity of FGR produces more pronounced effects in the fetal liver, with greater glucose output, higher gluconeogenic gene activation, lower glycogen, and increased markers of nutrient and energetic stress. As shown previously and herein, severity is associated with greater hypoxia (52) and higher lactate, pyruvate, and norepinephrine concentrations.

We acknowledge limitations and observations that warrant further investigation. Using U-^13^C-glucose tracer infusion in another fetal sheep model, we found significant plasma ^13^C-lactate (m+3) enrichment (53), which prompted us herein to measure plasma lactate enrichments and liver tissue ^13^C-metabolite labeling. We recognize that liver tissue ^13^C-labeling could be derived from the metabolism of m+6 glucose, m+3 lactate, or other ^13^C-labeled metabolites. Considering this, the ratio of the fractional labeling of m+3 lactate relative to m+6 glucose was greater than 1.0 (Supplemental Figure 1D), supporting higher uptake of labeled lactate relative to glucose. Further, the higher ratio of labeled hepatic lactate output relative to uptake (see Figure 4H) may reflect (a) increased lactate production within the FGR liver from ^13^C-labeled precursors, or (b) that lactate is simultaneously produced and utilized for HGP (i.e., intracellularly shuttling) without release and dilution of the h_out_/h_in_ labeling ratio. In addition, fetal (umbilical) blood exchanges across the placenta with maternal blood, and our fetal tracer infusion is based on an estimated fetal weight. This will produce differences in net tracer uptake that we account for by using ratios of labeled metabolites; however, this does not consider differences in the total abundance of a metabolite. Furthermore, differences in lactate production and oxidation in cytosol versus mitochondria would support intracellular lactate shuttling (46) and may affect the availability of lactate for HGP and redox balance (45). Thus, to address technical issues around ^13^C-labeling and better understand hepatic lactate metabolism, future studies are needed with different tracer approaches. We focused on the major substrates in the fetal liver. Other substrates, including glycerol and free fatty acids, can provide carbon and energy substrates to fuel HGP postnatally (30). In the fetal sheep, however, lipids provide little fuel for oxidative metabolism and free fatty acid concentration differences are not detectable across the umbilical or fetal hepatic circulation (15, 54). Net glycerol uptake by the fetus under normal conditions is quantitatively small (0.6 μmol/min/kg) and undetectable across the fetal liver even during hypoglycemia with active HGP (3, 55). Other secondary metabolites for gluconeogenesis, oxidation, or nutrient signaling could be altered during FGR, including acetate, propionate, keto-acids, acyl-carnitines, and lipid metabolites. Additional studies are needed to interrogate these metabolites. Lastly, adult sheep have an active rumen and digestive system that is different compared with humans; however, this digestive physiology is not active in the fetus, and glucose metabolism in fetal sheep is quite similar to that in humans under normal and FGR conditions (54, 56). Species differences for other substrates, including lipids, may exist in sheep compared with human fetuses.

In summary, the fetal liver receives the first pass at nutrients coming from the placenta, placing it in a critical position to respond to decreased nutrient and oxygen supply during placental insufficiency and resulting FGR. Our results identify increased intrahepatic lactate production, inhibition of pyruvate oxidation, and decreased glutamate output as pathways that make carbon substrates available to support increased HGP in the FGR fetus. Increased HGP will increase glucose supply for extrahepatic fetal tissues and increased hepatic pyruvate output may improve intrahepatic and systemic redox balance. These adaptive responses in the liver are advantageous for the FGR fetus; however, intrinsic programming may cause dysregulated HGP and T2D predisposition in postnatal FGR offspring. These results can be used in future studies to develop and test targeted effective prevention or intervention strategies aimed to improve neonatal glucose homeostasis or reduce later-life T2D risk, respectively, in FGR offspring.

## Methods

### Sex as a biological variable.

Our study included male and female fetuses, but was not powered to detect sex differences and data from female and male fetuses were combined. Our prior work has shown no differences in fetal growth or blood flow in male versus female fetuses from CON and FGR pregnancies (57), nor did we find differences in uterine or umbilical uptake rates in fetuses studied previously (15).

### Sheep model of FGR.

Pregnant Columbia-Rambouillet ewes with singleton pregnancies were supplied from Nebeker Ranch. Experimental work was performed and reported according to the Animal Research: Reporting of In Vivo Experiments guidelines (58). FGR fetuses were created by exposing pregnant ewes to elevated humidity and temperature (40°C for 12 hours, 35°C for 12 hours) from approximately 37 days gestation age (dGA, term = approximately 147 dGA) to approximately 116 dGA in an environmentally controlled room using our established model (4, 15, 57). CON fetuses were from pregnant ewes exposed to normal humidity and temperatures daily (25°C) and pair fed to the intake of the FGR ewes. After treatment, all ewes were exposed to normal humidity and temperatures until time of study. Surgery was performed in 6 CON and 10 FGR pregnancies at approximately 125 days of gestation (~147 dGA) to place indwelling catheters in the fetal vasculature (3, 15, 48). Briefly, ewes were fasted for 24 hours and administered diazepam (0.2 mg/kg) and ketamine (17.5 mg/kg) followed by inhalation anesthesia (isoflurane, 2% to 5%). Fetal catheters were placed in 1 of the 2 umbilical veins (advanced into the common umbilical vein), fetal artery (advanced into the abdominal aorta), brachial vein (advanced into the superior vena cava), and left hepatic vein representing blood leaving the liver (h_out_) (3). Catheters were filled with 5% heparinized saline, exteriorized, and kept in a plastic pouch sutured to the skin. The animals in this study were part of a study measuring uterine and umbilical nutrient flux where additional animal care, surgical, and analytical methods are described (15).

### Fetal hepatic blood flow and nutrient flux.

Following a minimum of 5 days postoperative recovery, studies were performed (~132 days) to measure fetal hepatic blood flow and oxygen and nutrient uptake rates using methods previously described (3). Infusates were prepared in a single syringe and infused into the fetal brachial vein. Indocyanine green (ICG) dye in saline plus 0.5% BSA was infused to measure hepatic blood flow (bolus: 2.1–2.9 mg; rate: 0.03–0.04 mg/min). A 3-mL bolus of ^3^H_2_O was infused followed by continuous infusion at 3 mL/hr (15 μCi/mL ^3^H_2_O) to estimate the contribution of umbilical venous and fetal arterial blood entering the left hepatic lobe (h_in_). To trace glucose metabolism, a [U-^13^C] glucose tracer was infused at 3 mL/hr (30 mg/mL [U-^13^C] glucose with 3 mL bolus). After 90–120 minutes, blood was simultaneously sampled from the umbilical vein, fetal artery, and left fetal hepatic vein 4 times at 20- to 30-minute intervals to characterize the steady-state period. Before the start of the tracer infusions, baseline blood draws were obtained for tracer background corrections. Fetal blood was replaced isovolumetrically with heparinized maternal arterial blood (15 mL/hr). Immediately following the study, ewes and fetuses were anesthetized with diazepam (0.2 mg/kg) and ketamine (17.5 mg/kg). Under these anesthetic conditions with all tracer infusions continuing, a piece of the left lobe of the liver was obtained and immediately flash-frozen. Subsequently, sodium pentobarbital (390 mg/mL, Fatal Plus) was administered to euthanize the ewe and fetus. For each fetus, weight was recorded, the liver was dissected, and weights of right and left lobes were obtained and portions were snap-frozen in liquid nitrogen. The location of the hepatic catheter within the left lobe was visually confirmed. The weight of the left liver tissue piece obtained under anesthesia was weighed after being frozen and summed with the weight of the remaining left lobe tissue for weight-based calculation. Hepatic or umbilical catheters did not draw for 1 CON and 2 FGR fetuses (one in moderate and one in severe weight group). Hepatic uptake data could not be calculated for the 3 fetuses, yet fetal weight, arterial concentrations, and liver tissue data were included. In addition, one FGR fetus (in moderate group) did not receive a ^13^C-glucose tracer infusion.

### Biochemical analyses.

Whole blood *p*O_2_ and O_2_ content, and plasma glucose, lactate, pyruvate, amino acid, and free fatty acid concentrations were measured as described previously (15).

### Hepatic blood flow and substrate uptake rates.

Left hepatic blood flow was estimated using ICG concentrations measured in plasma samples from umbilical vein, fetal artery, and left hepatic vein as previously described (3, 27). Blood flow to the left hepatic lobe was calculated by application of the Fick principle. ^3^H_2_O concentrations were measured (15) and used to estimate the contribution of umbilical venous blood and fetal arterial blood to the left hepatic lobe (25, 27). These fractional inputs were used to calculate the hepatic input (h_in_) concentrations for each substrate. Net hepatic uptake of oxygen, glucose, pyruvate, lactate, and individual amino acids by the left lobe of the fetal liver was calculated by application of the Fick principle as follows: substrate uptake = *F* × ([substrate]_hin_ – [substrate]_hout_), where *F* is hepatic blood or plasma flow (mL/min), [substrate]_hin_ is the substrate concentration of the blood or plasma entering the liver, and [substrate]_hout_ is the substrate concentration of the blood or plasma leaving the liver as measured in the left hepatic vein (3, 27). To calculate the rate of carbon uptake, the net substrate uptake rate was multiplied by the number of carbon atoms in that substrate. Nutrient-oxygen metabolic quotients were calculated for glucose, lactate, pyruvate, and amino acids (21, 31, 59). For each animal, data obtained from the h_out_ catheter was verified to have lower oxygen content, higher carbon dioxide, and higher glutamate concentrations compared with h_in_, supporting correct sampling location.

### Glucose and lactate tracer enrichments.

Glucose tracer enrichments (molar percent excess [MPE]) were measured as previously described (15, 44, 51). Briefly, glucose was derivatized for gas chromatography/mass spectrometry (GC/MS) analysis and glucose [U-^13^C] m+6 enrichment was monitored at *m/z* of 334/328 ratio and m+3 enrichment at *m/z* of 331/328 ratio. Glucose MPE was calculated as the difference in peak area ratios between unenriched (baseline) and enriched samples. Whole blood glucose concentrations were calculated from plasma as described previously (60) and used for calculations. Total hepatic glucose utilization was calculated as the product of hepatic blood flow and molar quantity of U-^13^C-glucose (m+6) taken up by the liver (h_in_ – h_out_) divided by h_in_ MPE, as previously described in hepatic studies in the canine (61). Molar quantities of labeled glucose, and lactate below, were calculated using the MPE multiplied by concentration (mM). Total HGP represents the sum of net hepatic glucose output and total hepatic glucose utilization, based on the assumption that glucose is provided by endogenous production and uptake from the placenta. The isotopic enrichment of ^13^CO_2_/^12^CO_2_ was measured using isotope ratio MS to calculate hepatic glucose oxidation rates (44, 53).

For lactate enrichments from the [U-^13^C] glucose tracer infusion, lactate was derivatized and lactate m+3 enrichment was monitored at an *m/z* ratio of 264/261 (53). Total hepatic lactate utilization was calculated as the product of hepatic blood flow and molar quantity of U-^13^C-lactate (m+3) taken by the liver (h_in_ – h_out_) divided by h_in_ MPE. Total hepatic lactate production represents the difference between total hepatic lactate utilization and net hepatic lactate uptake, assuming that lactate is provided from endogenous production and uptake from the placenta.

### Fetal cohorts for liver tissue and primary hepatocyte studies.

Liver tissue samples were used from the fetuses described above (*n* = 6 CON, 10 FGR) for all assays. Additional liver samples were used from a second cohort of CON (*n* = 7) and FGR (*n* = 11) fetuses (50) for hepatic gene expression, glycogen, PDH and LDH activity, and TBARS measurements. To obtain homogeneous samples, left liver tissue from each animal was ground in liquid nitrogen. For primary hepatocyte studies, many of the above fetuses were used in addition to CON and FGR fetuses from other ongoing research projects in our research facility. Fetal weights for the additional cohorts are shown in Supplemental Figure 3.

### Hepatic gene expression.

Total RNA was isolated from liver tissue and qPCR was performed as previously described (20, 51, 62, 63). A list of the genes measured is provided in Supplemental Table 3. Primers were used as previously reported (22, 48, 64) or newly designed to span introns and avoid alternative spliced variants using ovine gene sequences in NCBI. The geometric mean of 4 reference genes was calculated with *RPS15*, *B2M*, *HMBS*, and *SMA* and used to normalize qPCR results. Data are expressed relative to the mean of the CON group.

### Hepatic protein expression.

Whole-cell protein lysates were prepared from liver tissue and Western immunoblotting was performed as previously described (51). The antibodies used are listed in Supplemental Table 4 and were validated in ovine samples (4, 20, 23, 48). Equality of sample loading was measured using the Total Protein Stain (LI-COR). Protein bands were visualized and quantified with Image Studio (LI-COR). Antibody specificity was verified by the presence of a single band at the expected molecular weight. Bands for phosphorylated and total forms of a protein were verified to be of similar size based on migration in gels when blot images were aligned. Results were quantified and expressed as a ratio of phosphorylated to total protein. Data are presented relative to the mean of the CON group.

### Hepatic tissue metabolite measurements and ^13^C-labeling.

Relative quantification of metabolite abundance was performed utilizing UHPLC-MS on a Vanquish UHPLC coupled online to a Q Exactive high-resolution mass spectrometer (Thermo Fisher Scientific) as described previously (62, 65). Samples were extracted in ice-cold lysis extraction buffer (methanol/acetonitrile/water, 5:3:2). MS analysis and data elaboration were performed as described previously (66). Metabolite assignments were performed using MAVEN (67). Peak intensity values for liver metabolites were analyzed with MetaboAnalyst 5.0 (68). Normalized data, by median with data auto-scaling, were analyzed with multivariate PLS-DA. For pathway analysis, the top 50 VIP-ranked metabolites were used in the Pathway Analysis module with Human KEGG pathway term library. Selected metabolites were used to generate a heatmap grouped by broad metabolic function. Other metabolites and ratios were calculated using non-normalized data and analyzed with univariate *t* tests. Metabolite ^13^C-labeling was detected for glucose, lactate, citrate, and malate (Supplemental Figure 1A). To assess PDH flux, ratios of m+2 citrate to m+3 lactate abundance and m+5 citrate to m+3 malate were used (69). Pyruvate m+3 and oxaloacetate m+3 labeling were below detectable limits; lactate and malate were used as proxies, respectively, given their rapid exchange. Lactate production was assessed as the ratio of m+3 lactate to m+6 glucose. Fractional labeling was calculated by dividing the abundance of a specific isotopomer by the sum of the abundance for all isotopomers detected and used to assess the relative rate of intrahepatic lactate production or hepatic uptake of labeled lactate produced from extrahepatic tissues.

### Hepatic glycogen and amino acid concentrations.

Fetal liver glycogen content was measured as described previously (70) and amino acid content using HPLC and internal standards for absolute quantification (15). Results were normalized to tissue weight.

### PDH and LDH enzymatic activity and TBARS.

Enzymatic activity of PDH (MAK183, Sigma-Aldrich) and LDH (ab102526, Abcam) was measured as previously described (51, 53). TBARS content (700870, Cayman Chemical) was measured as described previously (62). Results were normalized to protein content.

### Primary fetal hepatocytes and glucose production assays.

Primary hepatocytes were isolated from normal late-gestation fetal sheep. Briefly, a portion of the right lobe of the fetal liver was perfused and digested with collagenase, and hepatocytes were separated by centrifugation (4, 71, 72). Hepatocytes were plated in DMEM with 1.1 mM glucose supplemented with 2 mM glutamine, 2.2 mM lactate, 1 mM pyruvate, 1× nonessential amino acids, 100 U/mL penicillin-streptomycin, 1 nM insulin, 100 nM dexamethasone, and 10% FBS on collagen-coated Primaria (BD Falcon) plates. After a 4-hour attachment period, cells were washed, and media were replaced with serum-free DMEM plus 0.2% BSA (SF media). The next day, hepatocytes were washed and incubated in glucose production media (phenol red–free and glucose-free DMEM with 10 mM HEPES and 0.369% NaHCO_3_) with various treatments at ambient oxygen conditions with 5% CO_2_ for 24 hours. Within each set of hepatocytes from CON (*n* = 15) or FGR (*n* = 17) fetuses, treatments were performed under basal and stimulated conditions with 500 nM dexamethasone and 100 μM cAMP (D+C) in the absence or presence of 2 mM sodium pyruvate, 20 mM sodium lactate, TrophAmine (20% vol/vol), and 2 mM glutamine. Glucose in the media (glucose oxidase assay) and protein content of cells (BCA assay) per well were measured. All treatments were performed in duplicate wells and average values were used within a set of hepatocytes. Additional studies were performed in a second set of hepatocytes from CON and FGR fetuses (with *n* = 2–4 per group) using 3-MPA, UK5099, 7ACC2, or oxamate under D+C-stimulated conditions with 2 mM pyruvate and 20 mM lactate, unless otherwise indicated.

### Statistics.

Data were analyzed by unpaired, 2-tailed Student’s *t* test, Welch’s *t* test, or Mann-Whitney *U* test, when variances were different between groups, or 1-sample Wilcoxon’s test using Prism 9.0 (GraphPad Software). The analysis used is indicated in the figures and tables. Within the FGR group, weight threshold differences comparing moderate (fetuses weighing >2 kg) versus severe (fetuses weighing <2 kg) FGR were analyzed by post hoc Student’s *t* test. For hepatocyte studies, a mixed model 2-way ANOVA with main effects of substrate treatment and group (CON or FGR), interaction effect, and random effect of hepatocyte preparation to account for repeated measures within each set of hepatocytes. Data are presented as mean ± SEM. Statistical differences are declared at a *P* value of 0.05 or less and statistical trends toward significance at a *P* value of 0.15 or less are shown.

### Study approval.

All animal procedures were approved by the Institutional Animal Care and Use Committee at the University of Colorado, which is accredited by the American Association for the Accreditation of Laboratory Animal Care International.

### Data availability.

Data are available in the paper’s supplemental material and in the Supporting Data Values file or from the corresponding author upon request.

## Author contributions

SRW analyzed data, wrote the main manuscript text, and prepared figures and tables. DW, ECE, and SRW performed experiments. RBW, PJR, and LDB assisted with identifying the hypotheses to be tested, experimental design, and data interpretation. AS assisted with data analysis. All authors approved the final version of the work submitted for publication.

## Supplementary Material

Supplemental data

Unedited blot and gel images

Supporting data values

## Figures and Tables

**Table 1:**
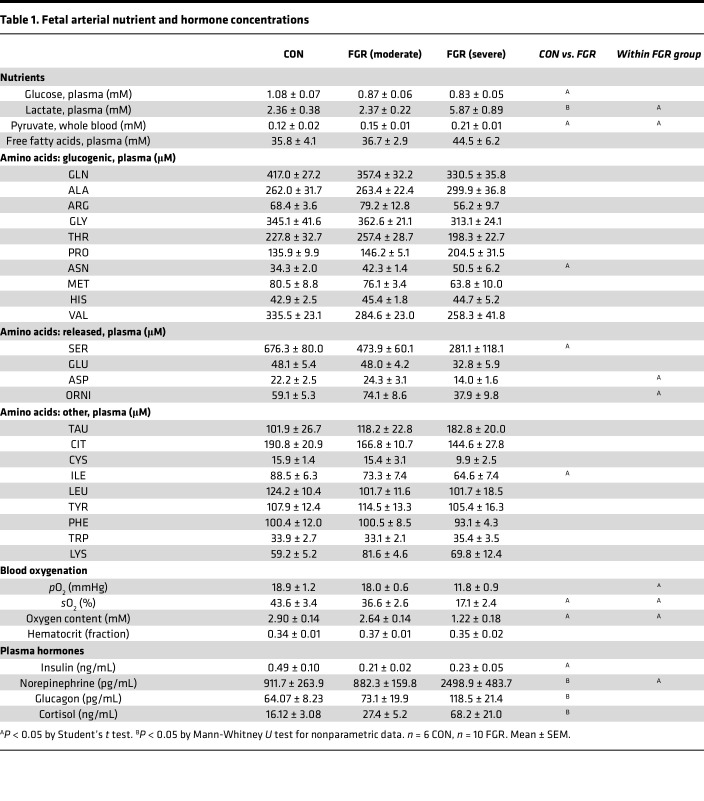
Fetal arterial nutrient and hormone concentrations

## Version 1 04/30/2024

In-Press Preview

## Version 2 05/22/2024

Electronic publication
