# Supplementary material for: Increased hepatic glucose production with lower oxidative metabolism in the growth-restricted fetus
Source: JCI Insight. 2024 Apr 30;9(10):e176497. doi: 10.1172/jci.insight.176497 (PMC11141920; doi:10.1172/jci.insight.176497)
Supplement: Supplemental data [file jciinsight-9-176497-s203.pdf]

Figure S1

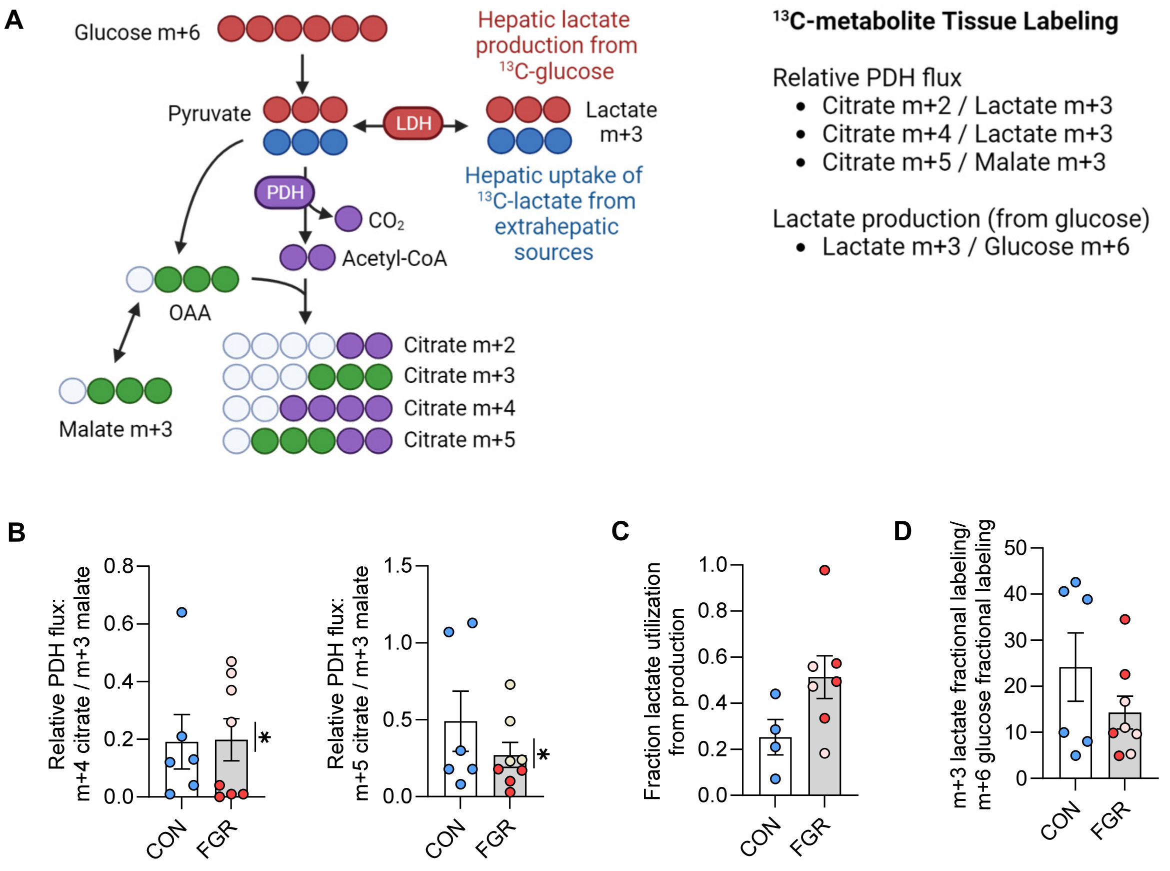

Figure S1. ^{13}C-metabolite tracing. A. Schematic of ^{13}C-metabolite tracing in liver tissue. ^{13}C-labeling in liver tissue could be derived from hepatic uptake of ^{13}C-glucose (red, m+6) or ^{13}C-lactate from extrahepatic tissues (blue, m+3). Within the liver, ^{13}C-glucose could be metabolized to ^{13}C-lacate (red, m+3) which would be indistinguishable from ^{13}C-lactate taken up by the liver (red) and would interconvert with pyruvate. Thus, the source of pyruvate labeling from endogenous production versus exogenous uptake can not be distinguished nor can the products of pyruvate metabolism to acetyl CoA via PDH (shown in purple) and oxaloacetate via PC (shown in green). To note, oxaloacetate labeling was undetectable. Thus, malate m+3 labeling was used as a proxy for oxaloacetate m+3 since malate equilibrates with oxaloacetate and is more abundant. B. PDH flux measured as the ratio of m+4 citrate relative to m+3 lactate, representing the incorporation after 2 rounds of the TCA cycle, and m+5 citrate relative to m+3 malate. C. Lactate production as a fraction of total lactate utilization. D. Ratio of m+3 lactate fractional labeling relative to m+6 glucose fractional labeling in liver tissue. *P<0.05, **P<0.01, ***P<0.001. Data points on graphs or blot lanes representing samples from FGR fetuses with moderate (pink) or severe (red) growth restriction are indicated. Within the FGR group, weight threshold differences comparing moderate (light pink) versus severe (dark red) FGR were analyzed by Student's t-test and are indicated by vertical line.

Figure S2

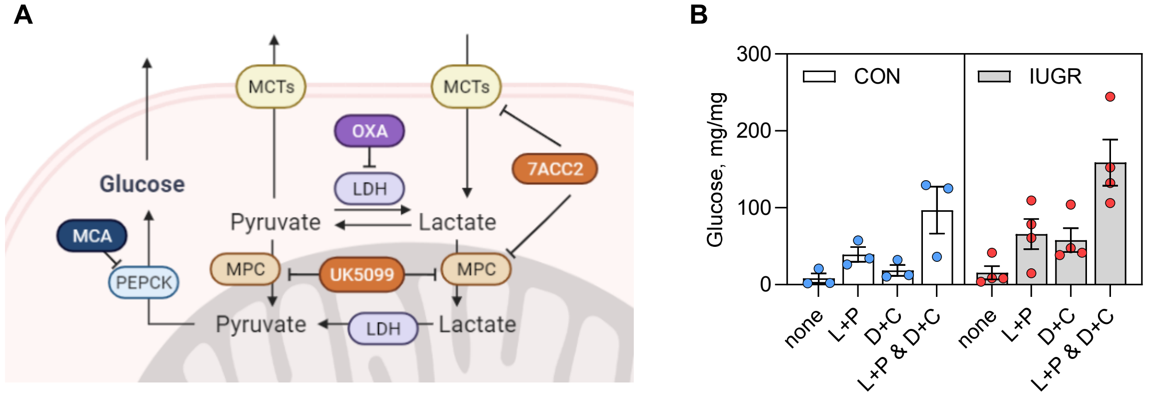

Figure S2. Glucose production studies with inhibitors. A. Pathways targeted by inhibitors used. B. Glucose production in isolated fetal primary hepatocytes from a second cohort of CON and FGR fetuses. L: lactate, P: pyruvate, D+C: dexamethasone and cAMP. 3-mercaptopicolinic acid (MCA),

Figure S3

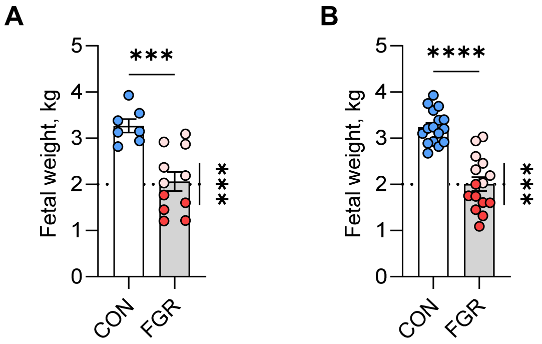

Figure S3. Fetal weights. A. Weights of additional fetuses that were used in tissue analyses. B. Weights of fetuses used for hepatocyte isolations and glucose production studies. Within the FGR group, weight threshold differences comparing moderate (light pink) versus severe (dark red) FGR were analyzed by Student's t-test and are indicated by vertical line.

Table S1. Metabolites in rank order by VIP scores.

Metabolite	Compound ID	General Category	VIP rank	VIP score	Q<0.20[a]
GDP	C00035	Nucleotides	1	2.125	*
NADPH	C00005	Nucleotides	2	2.1216	*
Putrescine	C00134	Polyamines	3	2.0445	*
ATP	C00002	Nucleotides	4	2.0285	*
gamma-L-Glutamylputrescine	C15699	Gamma-glutamyls	5	2.0061	*
L-lysine	C00047	Amino acids	6	1.9703	*
L-proline	C00148	Amino acids	7	1.8196	*
UDP	C00015	Nucleotides	8	1.7841	*
Phosphoenolpyruvate	C00074	Glycolysis	9	1.7717	*
UMP	C00105	Nucleotides	10	1.705	*
CDP	C00112	Nucleotides	11	1.6966	*
AMP	C00020	Nucleotides	12	1.6896	*
2/3-Phospho-D-glycerate	C00631	Glycolysis	13	1.6472	*
6-Phospho-D-gluconate	C00345	Pentose Phosphate Pathway	14	1.6442	*
L-glutamate	C00025	Amino acids	15	1.6366	*
S-Adenosyl-L-methionine	C00019	Serine biosynthesis and one-carbon metabolism	16	1.6349	*
Xanthine	C00385	Nucleotides	17	1.6073	*
NADH	C00004	Nucleotides	18	1.6001	*
L-valine	C00183	Amino acids	19	1.5985	*
Lactate	C01432	Glycolysis	20	1.5637	*
NADP+	C00006	Nucleotides	21	1.5552	*
ADP	C00008	Nucleotides	22	1.5441	*
L-alanine	C00041	Amino acids	23	1.524	*
2-Oxoglutarate	C00026	TCA cycle	24	1.4938	*
UDP-glucose	C00029	Nucleotides	25	1.4774	*
D-Fructose 1-6-bisphosphate	C00354	Glycolysis	26	1.4455	
Diphosphate	C00013	Phosphates	27	1.4272	*
CMP-N-acetylneuraminate	C00128	Aminosugars	28	1.3962	
Choline	C00114	Glycerophospholipid biosynthesis	29	1.3311	*
Urate	C00366	Nucleotides	30	1.3234	
Phosphocreatine	C02305	Arginine and proline metabolism	31	1.3165	
CDP-choline	C00307	Glycerophospholipid biosynthesis	32	1.2942	
Anthranilate	C00108	Indole and Tryptophan	33	1.2835	
Ethanolamine phosphate	C00346	Glycerophospholipid biosynthesis	34	1.27	
Citrate	C00158	TCA cycle	35	1.2548	
Succinate	C00042	TCA cycle	36	1.2454	
L-serine	C00065	Amino acids	37	1.2411	
NAD+	C00003	Nucleotides	38	1.2365	
5-6-Dihydrothymine	C00906	Nucleotides	39	1.2078	
Pyridoxal	C00250	Nucleotides	40	1.1448	*

Indolepyruvate	C00331	Indole and Tryptophan	41	1.1438
L-glutamine	C00064	Amino acids	42	1.1424
Adenine	C00147	Nucleotides	43	1.1397
gamma-L-Glutamyl-D-alanine	C03738	Gamma-glutamyls	44	1.1227
Glutathione	C00051	GSH homeostasis	45	1.1067
dAMP	C00360	Nucleotides	46	1.1012
Sphingosine 1-phosphate	C06124	Glycerophospholipid biosynthesis	47	1.0549
Phosphate	C00009	Phosphates	48	1.0507
Malate	C00149	TCA cycle	49	1.046
(5-L-Glutamyl)-L-glutamine	C05283	Gamma-glutamyls	50	1.0305
**Mannitol**	C00392	Other sugars	51	1.028
N-Glycoloyl-neuraminate	C03410	Aminosugars	52	1.0276
Glycerol 3-phosphate	C00093	Glycerophospholipid biosynthesis	53	1.0211
trans-4-Hydroxy-L-proline	C01157	Arginine and proline metabolism	54	1.0172
D-Glyceraldehyde 3-phosphate/Glycerone phosphate	C00118	Glycolysis	55	1.0053
L-phenylalanine	C00079	Amino acids	56	1.0008
Pantetheine	C00831	Panthothenate metabolism	57	0.99824
5-Hydroxyindoleacetate	C05635	Indole and Tryptophan	58	0.97257
Sphingosine	C00319	Glycerophospholipid biosynthesis	59	0.97101
Fumarate	C00122	TCA cycle	60	0.96652
N-Methylethanolamine phosphate	C01210	Glycerophospholipid biosynthesis	61	0.95851
UDP-N-acetyl-D-glucosamine	C00043	Aminosugars	62	0.91371
3-Sulfino-L-alanine	C00606	Sulfur metabolism	63	0.90172
Homocarnosine	C00884	Arginine and proline metabolism	64	0.88832
Oxaloacetate	C00036	TCA cycle	65	0.87267
L-tyrosine	C00082	Amino acids	66	0.86335
Pantothenol	C00864	Panthothenate metabolism	67	0.86232
Sedoheptulose 1-phosphate	C06222	Pentose Phosphate Pathway	68	0.81905
Flavin mononucleotide	C00061	Nucleotides	69	0.81569
Biliverdin	C00500	Hemolysis markers	70	0.80585
kynurenine	C00328	Indole and Tryptophan	71	0.80202
Hypoxanthine	C00262	Nucleotides	72	0.80167
Dehydroascorbate	C05422	GSH homeostasis	73	0.79745
L-Methionine S-oxide	C02989	Sulfur metabolism	74	0.79329
10-Formyltetrahydrofolate	C00234	Folate pool (One carbon metabolism)	75	0.79168
Argininosuccinate	C03406	Urea cycle	76	0.79032
Bilirubin	C00486	Hemolysis markers	77	0.77054
Cystathionine	C00542	Serine biosynthesis and one-carbon metabolism	78	0.76581
4-Pyridoxate	C00847	Nucleotides	79	0.74555
Dopamine	C03758	Signaling	80	0.7416
1-4-beta-D-Xylan	C02352	Aminosugars	81	0.74134

6-Hydroxykynurenic acid	C08480	Indole and Tryptophan	82	0.73036
L-cysteine	C00097	Amino acids	83	0.72213
L-aspartate	C00049	Amino acids	84	0.70449
5-Hydroxyisourate	C11821	Nucleotides	85	0.70016
Adenosine	C00212	Nucleotides	86	0.69891
L-asparagine	C00152	Amino acids	87	0.68573
Glutathione disulfide	C00127	GSH homeostasis	88	0.68536
N-Acetylornithine	C00437	Arginine and proline metabolism	89	0.68345
Phosphoserine	C02532	Serine biosynthesis and one-carbon metabolism	90	0.68281
Cys-Gly	C01419	GSH homeostasis	91	0.68068
Creatinine	C00791	Arginine and proline metabolism	92	0.67511
2-Oxoglutaramate	C00940	TCA cycle	93	0.67298
D-Rhamnose	C01684	Other sugars	94	0.65924
L-methionine	C00073	Amino acids	95	0.65089
L-Carnitine	C00318	Carnitine and fatty acid metabolism	96	0.64061
Inosine	C00294	Nucleotides	97	0.62131
Ornithine	C01602	Urea cycle	98	0.6092
L-threonine	C00188	Amino acids	99	0.60453
quinolinic acid	C03722	Indole and Tryptophan	100	0.60265
Thymidine	C00214	Nucleotides	101	0.6026
Adenylosuccinic acid	C03794	Nucleotides	102	0.58426
L-Citrulline	C00327	Urea cycle	103	0.58057
Guanine	C00242	Nucleotides	104	0.56195
Acetylcholine	C01996	Glycerophospholipid biosynthesis	105	0.55309
D-Glucosamine	C00329	Aminosugars	106	0.5471
GMP	C00144	Nucleotides	107	0.52132
glycine	C00037	Amino acids	108	0.50655
(S)(+)-Allantoin	C02350	Nucleotides	109	0.50143
Indole-3-acetaldehyde	C00637	Indole and Tryptophan	110	0.49691
Taurine	C00245	Sulfur metabolism	111	0.47429
Carnosine	C00386	Arginine and proline metabolism	112	0.47026
Guanosine	C00387	Nucleotides	113	0.43406
Allantoate	C00499	Nucleotides	114	0.41465
Hypotaurine	C00519	Sulfur metabolism	115	0.41463
Dimethylglycine	C01026	Serine biosynthesis and one-carbon metabolism	116	0.39239
L-tryptophan	C00078	Amino acids	117	0.3901
S-Adenosyl-L-homocysteine	C00021	Serine biosynthesis and one-carbon metabolism	118	0.38631
S-Glutathionyl-L-cysteine	C05526	GSH homeostasis	119	0.38509
1-3-Bisphosphoglycerate	C00236	Glycolysis	120	0.35447
L-arginine	C00062	Amino acids	121	0.35229
D-Arabitol	C01904	Other sugars	122	0.32336
5-Phospho-alpha-D-ribose 1-diphosphate	C00119	Pentose Phosphate Pathway	123	0.31836

CMP	C00055	Nucleotides	124	0.30785
Indole	C00463	Indole and Tryptophan	125	0.30199
Nicotinamide	C00153	Nucleotides	126	0.28805
5-L-Glutamyl-taurine	C05844	Gamma-glutamyls	127	0.28338
2-Hydroxyglutarate/Citramalate	C02630	Alternative Carboxylic acids	128	0.27217
N-Acetylneuraminate	C00270	Aminosugars	129	0.27011
ADP-D-ribose	C01882	Nucleotides	130	0.24588
3-Sulfocatechol	C06336	Sulfur metabolism	131	0.24387
IMP	C00130	Nucleotides	132	0.24184
alpha-D-Glucosamine 1-phosphate	C06156	Aminosugars	133	0.24103
Cytidine	C00475	Nucleotides	134	0.21616
gamma-L-Glutamyl-L-cysteine	C00669	Gamma-glutamyls	135	0.1976
L-histidine	C00135	Amino acids	136	0.19079
D-Glucose	C00031	Glycolysis	137	0.18856
Pyruvate	C00022	Glycolysis	138	0.16498
Creatine	C00300	Arginine and proline metabolism	139	0.15775
D-Glucono-1-5-lactone 6-phosphate	C01236	Pentose Phosphate Pathway	140	0.15606
Ectoine	C06231	Inositol	141	0.13064
D-Erythrose 4-phosphate	C00279	Pentose Phosphate Pathway	142	0.11558
alpha-D-Ribose 1-phosphate	C00620	Pentose Phosphate Pathway	143	0.1113
L-leucine/isoleucine	C00123	Amino acids	144	0.092879
Ascorbate	C00072	GSH homeostasis	145	0.07051
L-Homocysteine	C00155	Serine biosynthesis and one-carbon metabolism	146	0.07018
5-Oxoproline	C01879	GSH homeostasis	147	0.050747
D-Ribose	C00121	Other sugars	148	0.039192
4-Acetamidobutanoate	C02946	Arginine and proline metabolism	149	0.039022
gamma-Glutamyl-gamma-aminobutyrate	C15767	Gamma-glutamyls	150	0.027038
Cytosine	C00380	Nucleotides	151	0.0082154
D-Glucose 6-phosphate	C02965	Glycolysis	152	0.0080158

[a] FDR-adjusted p-value (Q-value) <0.20 indicated with *.

Table S2. Top 20 pathways enrichment among metabolites regulated in FGR fetal liver.

Metabolite Set[a]	Enrichment ratio	FDR adj-P-value	- log (FDR adj-P-vlaue)
Warburg Effect*	6.897	2.03E-10	9.69
Glutamate Metabolism*	6.364	1.87E-07	6.73
Arginine and Proline Metabolism	5.882	3.92E-07	6.41
Pyruvate Metabolism*	6.019	7.98E-07	6.10
Urea Cycle	7.692	2.27E-06	5.64
Nicotinate and Nicotinamide Metabolism	6.627	2.27E-06	5.64
Purine Metabolism	4.518	2.27E-06	5.64
Glucose-Alanine Cycle*	11.986	3.99E-06	5.40
Ammonia Recycling	6.944	4.14E-06	5.38
Phytanic Acid Peroxisomal Oxidation	7.692	5.80E-06	5.24
Gluconeogenesis*	6.369	8.73E-06	5.06
Glutathione Metabolism*	8.484	9.57E-06	5.02
Transfer of Acetyl Groups into Mitochondria	8.097	1.34E-05	4.87
Alanine Metabolism*	9.162	2.26E-05	4.65
Citric Acid Cycle*	6.250	2.74E-05	4.56
Phosphatidylethanolamine Biosynthesis	11.132	2.74E-05	4.56
Cysteine Metabolism*	6.838	4.04E-05	4.39
Ethanol Degradation	8.197	4.04E-05	4.39
Histidine Metabolism	5.181	4.04E-05	4.39
Phosphatidylcholine Biosynthesis	9.539	0.00007	4.18

[a] Pathways shown in Figure 3b are indicated (*).

Table S3. Primers for qPCR

Common gene name	Symbol	Forward Primer	Reverse Primer
glucose-6-phosphatase catalytic-subunit	*G6PC*	GGATTCTGGATCGTGCAACT	ATCCAATGGCGAAACTGAAC
phosphoenolpyruvate carboxykinase 1, cytosolic	*PCK1*	TGTCCGAGGAGGATTTTGAG	ATGCCAATCTTGGACAGAGG
phosphoenolpyruvate carboxykinase 2, mitochondrial	*PCK2*	GCCTGTGCTTCAGGCCCTGG	TGCATGGCCACTGGCACACC
Pyruvate carboxylase	*PC*	GCACAGCATGGGGCTTGGCT	AACTGGGCCAGGTCCCCCAC
glutaminase 1	*GLS1*	CCCAGAAGGCACAGACATGGTTGG	GGGCAGAAGCCACCATTAGCCA
glutaminase 2	*GLS2*	CTGGTGCCATTGTTGTGAGC	ATGTGGCATTGCTGAAACCC
glutamate dehydrogenase 1	*GLUD1*	AGCGCTCTGCCAGGCAAATCAT	GCGGCCGTTCTCAGGTCCAG
SLC1A5 solute carrier family 1 member 5	*SLC1A5*	GCACAGCTCAACCACCGGTCC	GCTCCAGATGGGATGCCCGC
Phosphoglycerate dehydrogenase	*PHGDH*	GGGAACCGAGCTGAATGGAA	GACCTCTGGCGAGATGATGG
phosphoserine aminotransferase 1	*PSAT1*	TCCAGGCCAGTGGATGTTTC	AACGATCACCACTGTGACCC
SLC1A5 solute carrier family 1 member 4	*SLC1A4*	CGGGCTGCCCACTCATGACC	GCCCTCCACGTTCACCACGG
Pyruvate dehydrogenaes kinase 1	*PDK1*	TGGAGCATCACGCTGACAAA	CTCAGAGGAACACCACCTCC
Pyruvate dehydrogenase kinase 4	*PDK4*	CCCAGAGGACCAAAAGGCAT	GGGTCAGCTGTACAGGCATC
Lactate dehydrogenase A	*LDHA*	CATGGCCTGTGCCATCAGTA	GGAAAAGGCTGCCATGTTGG
Lactate dehydrogenase B	*LDHB*	GAGGGAGCGATCCCAAACAA	CAGAATGCTGATGGCACACG
Solute carrier family 16 member 1	*MCT1*	GTGGCTTGATTGCTGCTTCC	GCCAATCATGGTCAAAGCCG
Solute carrier family 16 member 4	*MCT4*	TTCTTTGCTGGGATGGCTGT	AGGAGATAGCATGCTCCCGA
Mitochondrial pyruvate carrier, 2	*MPC2*	TAAAGTGGAGCTCCTGCTGC	ATGTCAGCCAATCCAGCACA
Mitochondrial pyruvate carrier, 1	*MPC1*	TCGGAACTGGCTCCTGTTTG	GCCGGTTCTTCATCTCCCAT
PPARG coactivator 1 alpha	*PGC1A*	GTGACTCTGGGGTCAGAGGA	CACCAAACCCACAGAGAACC
Serum/glucocorticoid regulated kinase 1	*SGK1*	ACCGTGGACTGGTGGTGCCT	TGGCTTCAGCTGGAGGGGCT
MYC proto-oncogene, bHLH transcription factor	*MYC*	CCCCTGCCAAAAGGTCAGAA	CTTTAGGACCAACGGGCTGT
Lipin-1	*LPIN1*	AGGAGAGCTTGTGCAGGAAC	GGGGAAGTCAGAAGAGTGGC
Ribosomal proten S15	*S15*	ATCATTCTGCCCGAGATGGTG	CGGGCCGGCCATGCTTTACG
beta-2-microglobulin	*B2M*	CTTGGTCCTTCTCGGGCTG	ATCTTCTGGCGGGTGTCTTG
hydroxymethylbilane synthase	*HMBS*	GTTCCCACACCTGGAGTTCA	CCTGCAGCTCATCCAGCTTA
smooth muscle actin	*SMA*	GAAGATCCTGACCGAGCGAG	TCGTTCTCAAAGTCCAGGGC

Table S4. Antibodies

Antibody	Dilution	Supplier (Catalog #)
ph-AMPK (Thr172)	1:1000	Cell Signaling (2531S)
AMPK alpha	1:1000	Cell Signaling (2793S)
P-SAPK/JNK (T183/Y185)	1:1000	Cell Signaling (9251)
JNK	1:1000	Cell Signaling (9252)
ph-FOXO1 (T24)/FOXO3a (T32)	1:1000	Cell Signaling (9464)
FOXO1 (L27)	1:500	Cell Signaling (9454)
ph-AKT (Ser473)	1:1000	Cell Signaling (4060)
AKT	1:1000	Cell Signaling (2920)
NRF2	1:1000	Invitrogen (PA5-27882)
