# Supplementary material for: Increased hepatic glucose production with lower oxidative metabolism in the growth-restricted fetus
Source: JCI Insight. 2024 Apr 30;9(10):e176497. doi: 10.1172/jci.insight.176497 (PMC11141920; doi:10.1172/jci.insight.176497)
Supplement: Unedited blot and gel images [file jciinsight-9-176497-s204.pdf]

AMPK – blot DW39

pAMPK (62KD)

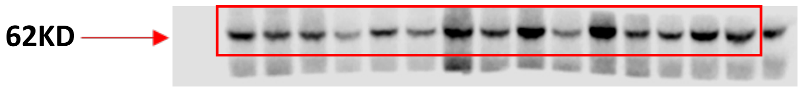

tAMPK

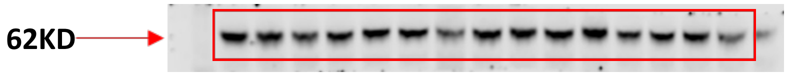

The bands in ☐ are quantified

JNK – blot DW38

P-JNK (46,54KD)

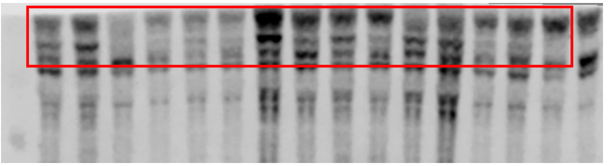

t-JNK

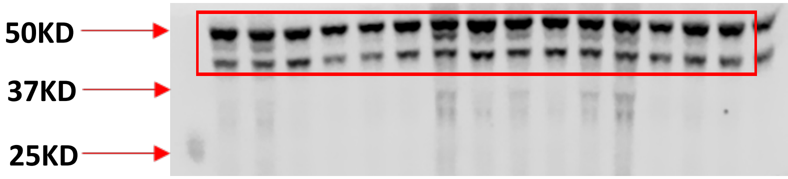

The bands in ☐ are quantified

FOXO1 – blot DW38

P-FOXO1 (78-82,85KD)

75KD

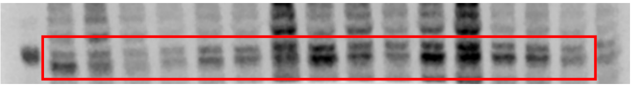

t-FOXO1

75KD

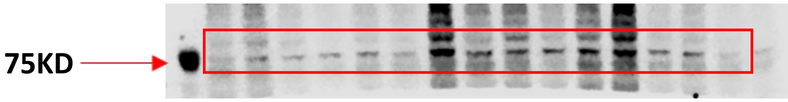

The bands in ☐ are quantified

NRF2 – blot DW224

NRF2 (MW 90KD)

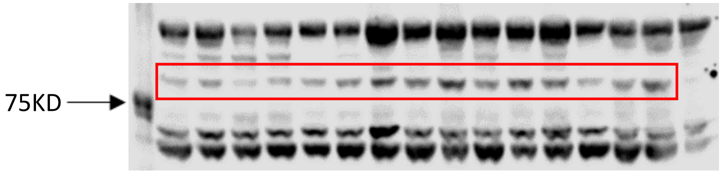

The bands in ☐ are quantified

Actin – blot DW39

ACTB (42KD)

DW39

42KD

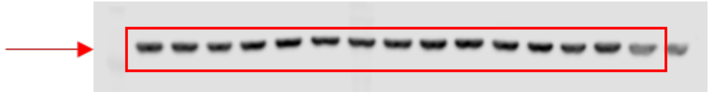

DW40

The bands in ☐ are quantified
